# Long-term oncologic outcomes associated with clinically significant anastomotic leakage after curative colorectal cancer surgery: Stage- and location-stratified analyses

**DOI:** 10.1371/journal.pone.0359416

**Published:** 2026-10-01

**Authors:** Youngbae Jeon, Sun Jin Sym, Won-Suk Lee, Jeong-Heum Baek

**Affiliations:** 1 Division of Colon and Rectal surgery, Department of Surgery, Gil Medical Center, Gachon University College of Medicine, Incheon, Republic of Korea; 2 Department of Medical Oncology, Gil Medical Center, Gachon University College of Medicine, Incheon, Republic of Korea; E-Da Cancer Hospital, TAIWAN

## Abstract

**Background:**

Clinically significant anastomotic leakage (AL) after colorectal cancer surgery is associated with substantial postoperative morbidity, but its long-term oncologic impact remains uncertain. We evaluated the association of AL with long-term survival, including stage- and location-specific patterns, after curative colorectal cancer surgery.

**Methods:**

We retrospectively reviewed consecutive patients who underwent curative surgery for non-metastatic colorectal adenocarcinoma between 2004 and 2018. Clinically significant AL was defined as grade B or C leakage. Overall survival (OS) and disease-free survival (DFS) were assessed using Kaplan–Meier and multivariable Cox regression analyses. Subgroup and formal interaction analyses were performed according to tumor stage and location.

**Results:**

Among 2,122 patients, 63 (3.0%) developed clinically significant AL. After multivariable adjustment, including treatment era, AL was not independently associated with OS (HR, 1.581; 95% CI, 0.923–2.707; p = 0.095) or DFS (HR, 1.325; 95% CI, 0.812–2.161; p = 0.260). In stage III disease, patients with AL had lower 5-year OS (56.3% vs. 76.3%; p = 0.005) and DFS (43.9% vs. 68.4%; p = 0.007). However, formal interaction testing showed no significant effect modification by tumor stage (OS, p for interaction = 0.132; DFS, p for interaction = 0.141) or tumor location (OS, p for interaction = 0.775; DFS, p for interaction = 0.748).

**Conclusions:**

Clinically significant AL was not independently associated with long-term oncologic outcomes in the overall cohort. Although poorer survival was observed in the stage III subgroup, the absence of significant interaction indicates that these stage- and location-specific findings should be interpreted as exploratory rather than evidence of differential AL effects.

## Introduction

Colorectal cancer is the third most commonly diagnosed malignant disease, with the second highest mortality rate worldwide [1]. The outcome of colorectal cancer surgery has significantly improved over the last few decades due to the advancements in surgical techniques, use of prophylactic antibiotics, and implementation of multimodal perioperative care strategies [2,3]. Nevertheless, anastomotic leakage (AL) remains one of the most serious complications following colorectal cancer surgery, with a pooled incidence of approximately 8.6% reported in a meta-analysis of more than 10,000 patients [4]. Several studies reported that the incidence rates of AL in colorectal cancer surgery are approximately 2–19% [5], and can increase up to 30%, especially with rectal cancer procedures [6–8]. AL usually results in peritonitis and might require antibiotic therapy, interventions, reoperation, or even stoma diversion. Such complications prolong hospital stay and negatively affect the patient’s quality of life. Although there is a consensus that AL after colorectal cancer surgery adversely affects short-term clinical outcomes, including postoperative morbidity and mortality [9,10], its impact on long-term oncologic outcomes remains inconsistent. Some studies have demonstrated that AL has a statistically significant negative impact on overall survival and cancer-specific survival [11,12], whereas others have found no significant differences in survival outcomes [13,14]. Previous studies on the long-term oncologic impact of AL have predominantly focused on rectal cancer, whereas relatively fewer studies have specifically evaluated patients with colon cancer [10,15–17]. Furthermore, studies investigating the stage-specific survival impact of AL are lacking. Therefore, in this study, we aimed to evaluate the long-term oncologic impact of AL in patients with non-metastatic colorectal adenocarcinoma who underwent curative surgery using a 15-year database, classified by tumor location (colon vs. rectum) and tumor-node-metastasis (TNM) stage.

## Methods

### Patient selection and ethics statement

This study included patients who underwent curative surgery for colorectal adenocarcinoma between January 2004 and December 2018 in Gil Medical Center, a tertiary referral hospital in South Korea. Patients with colon cancer underwent radical colectomy with D2 lymph node dissection, whereas those with rectal cancer underwent total mesorectal excision with D2 lymph node dissection. In selected patients of locally advanced mid to low rectal cancer, long-course neoadjuvant chemoradiotherapy (NCRT), including a total dose of 50.4 Gy of external beam radiation, with intravenous fluorouracil or oral capecitabine, was performed 6–10 weeks before surgery after tumor board discussion. Exclusion criteria were as follows: histological types other than colorectal adenocarcinoma; patients aged ≤18 years; metastatic, recurrent, and hereditary colorectal cancers; patients diagnosed with other cancers within 5 years before surgery; incomplete surgery (R1 or R2 resection); surgeries without anastomosis (e.g., Hartmann procedure, abdominoperineal resection, or transanal excision); and emergency surgery. Patient selection was based on the Clinical Research Data Warehouse system, and data were collected from the electronic medical records at Gil Medical Center. The data were accessed for research purpose since September 19, 2023. All data has been anonymized so that individual participant could not be identified. Fig 1 shows a flowchart of the patient selection process (Fig 1).

**Fig 1:**
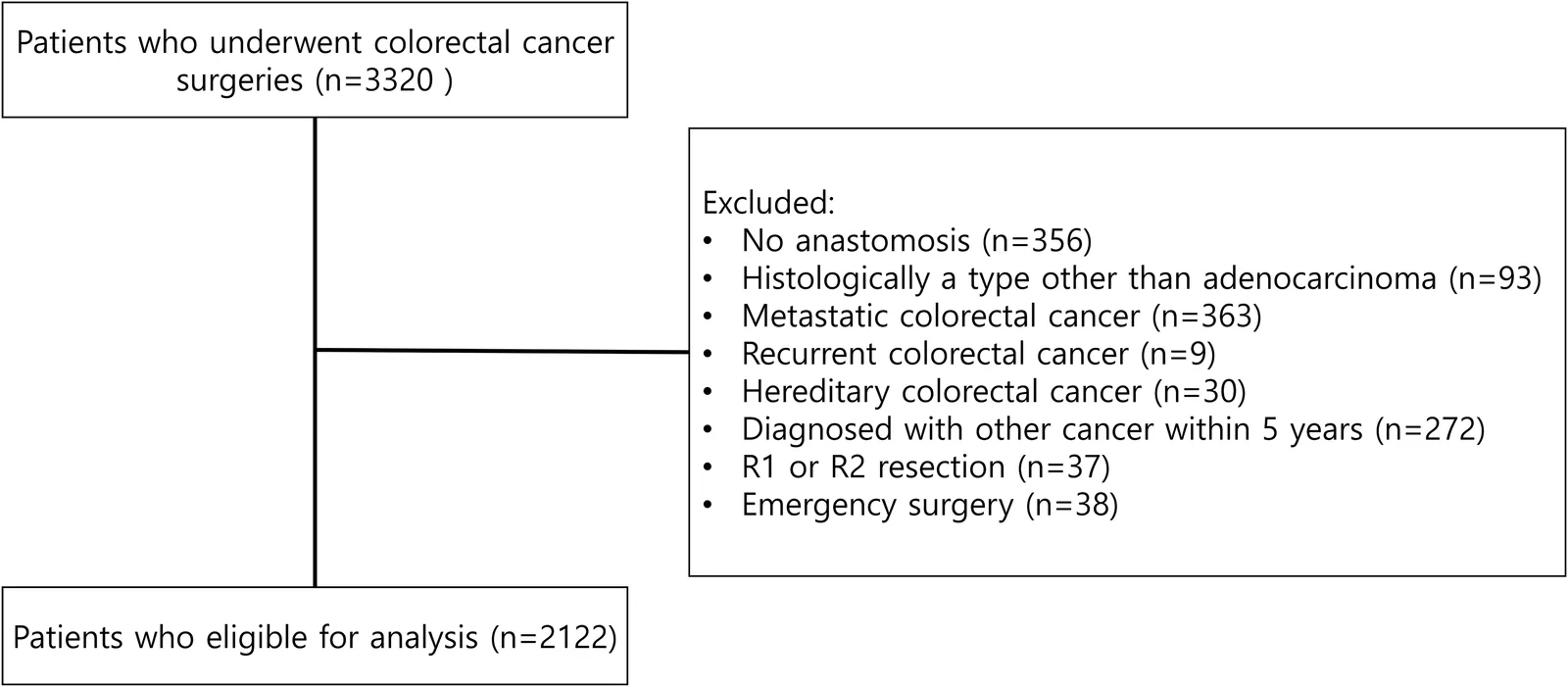
Flowchart of the patient selection process.

This study was approved by the Institutional Review Board of Gil Medical Center of Gachon University (approval no. GAIRB2023−331). The need for informed consent was waived due to the study’s retrospective design.

### Patient follow-up

Patients were followed up every 3–6 months for 5 years after curative surgery, with or without adjuvant chemotherapy. Follow-up evaluations included physical examination, serum carcinoembryonic antigen (CEA) level, abdominopelvic computed tomography (APCT), and chest CT. Colonoscopies were scheduled one year after surgery, and biennially thereafter. Additional imaging studies such as transrectal ultrasound (TRUS), pelvic magnetic resonance imaging (MRI), and positron emission tomography (PET) were conducted at the physician’s discretion.

### Assessment parameters and statistical analysis

The key variables included patient demographics and perioperative, histopathologic, and survival data. TNM staging was performed according to the seventh edition of the American Joint Committee on Cancer (AJCC) [18]. Surgical approaches included open and laparoscopic procedures, with cases converted from laparoscopic to open surgery classified as open surgery. The follow-up duration was defined as the time from primary resection to either death or the last recorded observation. Overall survival (OS) was calculated from the date of curative surgery to all-cause death. Disease-free survival (DFS) was calculated from the date of curative surgery to recurrence or all-cause death. AL in this study was characterized by peritonitis due to leakage, abscess formation near the anastomotic site, or the presence of fecal content and/or pus in the abdominal drain. Diagnosis of AL was confirmed using one or more of the following methods: physical examination, APCT, sigmoidoscopy, diagnostic laparoscopy, or laparotomy. This study included only clinically relevant leakage, excluding subclinical leakage detected only using imaging. Therefore, only AL grades B and C, according to the International Study Group of Rectal Cancer [19], were included in this study (Table 1). The grading system is typically used for rectal cancer; however, in this study, its relevance was extended to colon cancer. Patients were classified based on the presence of AL into anastomotic leakage (ALG) and no anastomotic leakage groups (NALG).

**Table 1:** Severity grading of anastomotic leakage (AL) after colorectal surgery.

Variables	Definition
Grade A	AL requiring no active therapeutic intervention
Grade B^a^	AL requiring active therapeutic intervention, but manageable without relaparotomy
Grade C^a^	AL requiring re-laparotomy

In this study, application of the AL grading system by the International Study Group of Rectal Cancer was extended beyond rectal cancer to include colon cancer.

^a^Only grades B and C were included in the analysis.

Categorical variables were compared between the ALG and NALG using Pearson’s chi-square test or Fisher’s exact test, as appropriate according to the expected cell counts. Clinically relevant variables, including age, sex, body mass index (BMI), American Society of Anesthesiologists (ASA) score, tumor location, histologic differentiation, and TNM stage were selected based on literature and included in univariate analysis. To account for temporal changes in surgical techniques, perioperative management, and oncologic treatment, the year of surgery was categorized into three 5-year treatment eras: 2004–2008, 2009–2013, and 2014–2018. Variables with p < 0.2 in the univariate analysis were entered into the multivariable Cox regression model. Clinically significant AL and treatment era were included regardless of their univariate significance, considering AL as the primary variable of interest and treatment era as a potential temporal confounder. Age was categorized as <70 and ≥70 years based on our institutional practice for preoperative risk assessment, in which patients aged ≥70 years routinely undergo additional cardiology and pulmonology evaluation regardless of underlying cardiopulmonary comorbidities. This cutoff is also consistent with previous studies and recommendations that have commonly used approximately 70 years to characterize older patients with colorectal cancer [20,21]. BMI was categorized as <25 and ≥25 kg/m², with BMI ≥ 25 kg/m² defined as obesity according to the Korean Society for the Study of Obesity guidelines [22]. Operative time was categorized as <150 and ≥150 minutes, using the median operative time of the study population as the cutoff. Hypoalbuminemia was defined as a serum albumin level of < 3.5 g/dL within 30 days before surgery.

Rectal cancer patients who received NCRT were included in the analysis, and receipt of NCRT was recorded as a clinicopathologic variable. For the stage-specific analyses, postoperative pathologic stage was used. Pathologic TNM stage (pTNM) was applied to patients who underwent upfront surgery, whereas post-neoadjuvant pathologic TNM stage (ypTNM) was applied to patients who received NCRT. Because NCRT was applicable only to rectal cancer, its association with long-term outcomes was evaluated within the rectal cancer subgroup rather than in the overall colorectal cancer cohort.

Stage 0 comprised patients with pTisN0M0 disease after upfront surgery and those with ypT0N0M0 disease after NCRT. Because of the small sample size and clinical heterogeneity of the stage 0 group, stage-specific survival analyses were restricted to stage I–III.

Survival analysis was performed using Kaplan-Meier method and log-rank test. Formal interaction analyses were performed using multivariable Cox proportional hazards models to evaluate whether the association between clinically significant AL and survival outcomes differed according to tumor stage or tumor location. Interaction terms between AL and tumor stage (AL × stage) and between AL and tumor location (AL × location) were evaluated separately for OS and DFS. Significant differences in each variable between the two groups were defined as p-values < 0.05. All statistical analyses were performed using the IBM SPSS Statistics version 23 for Windows (IBM Corp., Armonk, NY, USA).

## Results

### Anastomotic leakage

Among the 3320 patients initially selected, 2122 were eligible for analysis. Of those patients included in the analysis, 1,351 (63.7%) had colon cancer and 771 (36.3%) had rectal cancer. Clinically significant AL (grade B = 18 or C = 45) occurred in 63 patients (3.0%), including 23 patients with colon cancer and 40 patients with rectal cancer. Of the 23 patients with colon cancer who developed AL, 7 patients had grade B and 16 patients had grade C AL, whereas among the 40 patients with rectal cancer who developed AL, 11 patients had grade B and 29 patients had grade C AL. Overall, 18 patients (28.6%) had grade B AL and 45 (71.4%) had grade C AL.

### Patient demographics

The baseline characteristics of the patients are summarized in Table 2. There were no significant differences between the ALG and NALG in age, sex, BMI, American Society of Anesthesiologists (ASA) score, cardiovascular disease, diabetes mellitus, chronic kidney disease, hypoalbuminemia, or bowel obstruction. Among patients with rectal cancer, the proportion receiving neoadjuvant chemoradiotherapy was also comparable between the two groups (30.0% vs. 28.7%, p = 0.863). However, the ALG had a significantly higher proportion of rectal tumors than the NALG (63.5% vs. 35.5%, p < 0.001). The distribution of treatment era also differed significantly between the groups (p = 0.007), with a higher proportion of patients in the ALG undergoing surgery during 2014–2018 compared with the NALG (60.3% vs. 42.5%).

**Table 2:** Baseline patient characteristics.

Variables	ALG (n = 63), n (%)	NALG (n = 2059), n (%)	p^a^
Age, years			0.919
≥70	21 (33.3)	699 (33.9)	
<70	42 (66.7)	1360 (66.1)	
Sex			0.086
Male	45 (71.4)	1250 (60.7)	
Female	18 (28.6)	809 (39.3)	
BMI ≥ 25, kg/m^2^			0.608
Yes	18 (28.6)	651 (31.6)	
No	45 (71.4)	1408 (68.4)	
ASA score			0.421
1	10 (15.9)	242 (11.8)	
2	47 (74.6)	1672 (81.2)	
3	6 (9.5)	145 (7.0)	
Cardiovascular disease			0.117
Yes	34 (54.0)	906 (44.0)	
No	29 (46.0)	1153 (56.0)	
Diabetes mellitus			0.709
Yes	14 (22.2)	418 (20.3)	
No	49 (77.8)	1641 (79.7)	
Chronic kidney disease			0.585
Yes	1 (1.6)	28 (1.4)	
No	62 (98.4)	2031 (98.6)	
Serum albumin<3.5, g/dL			0.160
Yes	4 (6.3)	251 (12.2)	
No	59 (93.7)	1808 (87.8)	
Bowel obstruction			0.146
Yes	9 (14.3)	184 (8.9)	
No	54 (85.7)	1875 (91.1)	
Tumor location			<0.001^*^
Colon	23 (36.5)	1328 (64.5)	
Rectum	40 (63.5)	731 (35.5)	
NCRT^b^			0.863
Yes	12/40 (30.0)	210/731 (28.7)	
No	28/40 (70.0)	521/731 (71.3)	
Treatment era, year			0.007^*^
2004 ~ 2008	13 (20.6)	422 (20.5)	
2009 ~ 2013	12 (19.0)	762 (37.0)	
2014 ~ 2018	38 (60.3)	875 (42.5)	
Anastomotic leakage grade			n/a
Grade B	18 (28.6)	n/a	
Grade C	45 (71.4)	n/a	

ALG, Anastomotic leakage group; NALG, No anastomotic leakage group; ASA, American Society of Anesthesiologists; BMI, body mass index; NCRT, neoadjuvant chemoradiotherapy

* Statistical significance, p < 0.05.

^a^P values were calculated using Pearson’s chi-square test or Fisher’s exact test, as appropriate.

^b^NCRT was applicable only to patients with rectal cancer; therefore, the corresponding numbers and percentages were calculated among rectal cancer patients only.

### Perioperative outcomes

Perioperative outcomes are shown in Table 3. Surgical procedures differed significantly between the groups, with the ALG undergoing low anterior resection more frequently than the NALG (74.6% vs. 46.1%, p < 0.001). Operative time ≥150 minutes was more frequent in the ALG than in the NALG, although the difference was not statistically significant (61.9% vs. 51.5%, p = 0.103). Hospital stays of ≥10 days were significantly more frequent in the ALG (79.4% vs. 39.9%, p < 0.001). The 90-day mortality rates did not differ significantly between the groups (1.6% vs. 0.9%, p = 0.437).

**Table 3:** Perioperative outcomes.

Variables	ALG (n = 63), n (%)	NALG (n = 2059), n (%)	p^a^
Type of surgery			<0.001^*^
Right hemicolectomy	10 (15.9)	393 (19.1)	
Transverse colectomy	1 (1.6)	32 (1.6)	
Left hemicolectomy	1 (1.6)	70 (3.4)	
Anterior resection	4 (6.3)	569 (27.6)	
Low anterior resection	47 (74.6)	950 (46.1)	
Total colectomy	0 (0.0)	45 (2.2)	
Surgical approach			0.203
Open	19 (30.2)	479 (23.3)	
Laparoscopic	44 (69.8)	1580 (76.7)	
Time of surgery ≥150 min			0.103
Yes	39 (61.9)	1060 (51.5)	
No	24 (38.1)	999 (48.5)	
Anastomosis method			0.744
Handsewn	3 (4.8)	85 (4.1)	
Stapled	60 (95.2)	1974 (95.9)	
Length of hospital stay ≥10 days			<0.001^*^
Yes	50 (79.4)	822 (39.9)	
No	13 (20.6)	1237 (60.1)	
90-day mortality	1 (1.6)	18 (0.9)	0.437

* Statistical significance, p < 0.05.

^a^P values were calculated using Pearson’s chi-square test or Fisher’s exact test, as appropriate.

### Histopathologic outcomes

Histopathologic findings are presented in Table 4. No significant differences were observed between the two groups regarding histologic grade, TNM staging, lymphovascular and perineural invasion, or number of harvested lymph nodes.

**Table 4:** Histopathologic outcomes.

Variables	ALG (n = 63), n (%)	NALG (n = 2059), n (%)	p^a^
Histologic grade			1.000
WD/MD	62 (98.4)	2016 (97.9)	
PD	1 (1.6)	43 (2.1)	
Pathologic staging according to AJCC 7th edition			
T stage			0.974
T0/Tis/T1/T2	22 (34.9)	715 (34.7)	
T3/T4	41 (65.1)	1344 (65.3)	
N stage			0.586
N0	38 (60.3)	1311 (63.7)	
N1/N2	25 (39.7)	748 (36.3)	
TNM stage			0.663
Stage 0^b^	2 (3.2)	49 (2.4)	
pTisN0M0	1 (1.6)	25 (1.2)	
ypT0N0M0	1 (1.6)	24 (1.2)	
Stage Ⅰ	19 (30.2)	556 (27.0)	
Stage Ⅱ	17 (27.0)	709 (34.4)	
Stage Ⅲ	25 (39.7)	745 (36.2)	
Lymphovascular invasion			0.312
Yes	27 (42.9)	754 (36.6)	
No	36 (57.1)	1305 (63.4)	
Perineural invasion			0.317
Yes	13 (20.6)	328 (15.9)	
No	50 (79.4)	1731 (84.1)	
Harvested LN < 12			0.578
Yes	9 (14.3)	349 (16.9)	
No	54 (85.7)	1710 (83.1)	

AJCC, American Joint Committee on Cancer; LN, lymph node; WD/MD, well or moderately differentiated; PD, poorly differentiated

^a^P values were calculated using Pearson’s chi-square test or Fisher’s exact test, as appropriate.

^b^Stage 0 comprised patients with pTisN0M0 disease after upfront surgery and those with ypT0N0M0 disease after neoadjuvant chemoradiotherapy.

### Multivariate analysis

Multivariate Cox regression analysis identified several independent predictors of survival (Table 5). For OS, age ≥ 70 years (HR, 3.374; 95% CI, 2.730–4.170; p < 0.001), male sex (HR, 1.494; 95% CI, 1.209–1.846; p < 0.001), diabetes mellitus (HR, 1.283; 95% CI, 1.004–1.641; p = 0.047), bowel obstruction (HR, 1.496; 95% CI, 1.052–2.127; p = 0.025), poorly differentiated histology (HR, 2.378; 95% CI, 1.440–3.930; p < 0.001), advanced pathologic T stage (HR, 1.422; 95% CI, 1.094–1.849; p = 0.008), and advanced pathologic N stage (HR, 1.706; 95% CI, 1.367–2.129; p < 0.001) were independently associated with poorer survival. Treatment era was also independently associated with OS (overall p < 0.001); compared with patients treated during 2004–2008, those treated during 2014–2018 had a lower risk of death (HR, 0.514; 95% CI, 0.361–0.732; p < 0.001). For DFS, age ≥ 70 years (HR, 2.487; 95% CI, 2.050–3.016; p < 0.001), male sex (HR, 1.356; 95% CI, 1.119–1.644; p = 0.002), rectal tumor location (HR, 1.277; 95% CI, 1.048–1.556; p = 0.016), poorly differentiated histology (HR, 2.229; 95% CI, 1.402–3.544; p < 0.001), advanced pathologic T stage (HR, 1.708; 95% CI, 1.332–2.191; p < 0.001), advanced pathologic N stage (HR, 1.848; 95% CI, 1.508–2.265; p < 0.001), perineural invasion (HR, 1.370; 95% CI, 1.068–1.757; p = 0.013), and harvesting of fewer than 12 lymph nodes (HR, 1.415; 95% CI, 1.113–1.798; p = 0.005) were independently associated with poorer outcomes. Treatment era was also independently associated with DFS (overall p = 0.008), with patients treated during 2014–2018 having a lower risk of recurrence or death than those treated during 2004–2008 (HR, 0.629; 95% CI, 0.468–0.846; p = 0.002). Clinically significant AL was not independently associated with either OS (HR, 1.581; 95% CI, 0.923–2.707; p = 0.095) or DFS (HR, 1.325; 95% CI, 0.812–2.161; p = 0.260).

**Table 5:** Univariate and multivariate analysis for overall and disease-free survival in colorectal cancer.

Variables	Overall survival				Disease-free survival			
	Univariate log-rank test	Multivariate Cox regression analysis^a^			Univariate log-rank test	Multivariate Cox regression analysis^a^		
	*p*	HR	95% CI	*p*	*p*	HR	95% CI	*p*
Age ≥ 70, years	<0.001	3.374	2.730–4.170	<0.001^*^	<0.001	2.487	2.050–3.016	<0.001^*^
Male sex	0.003	1.494	1.209–1.846	<0.001^*^	0.006	1.356	1.119–1.644	0.002^*^
BMI ≥ 25, kg/m^2^	0.053	0.865	0.686–1.089	0.216	0.054	0.880	0.714–1.083	0.228
ASA > 1	<0.001	1.277	0.833–1.958	0.262	0.002	1.145	0.802–1.635	0.458
Cardiovascular disease	0.001	1.063	0.856–1.322	0.579	0.010	1.107	0.910–1.347	0.309
Diabetes Mellitus	0.006	1.283	1.004–1.641	0.047^*^	0.227			
Chronic kidney disease	0.009	1.604	0.818–3.145	0.169	0.025	1.440	0.761–2.726	0.262
Serum albumin<3.5, g/dL	0.803				0.627			
Bowel obstruction	<0.001	1.496	1.052–2.127	0.025^*^	0.002	1.277	0.922–1.770	0.141
Rectum	0.788				0.117	1.277	1.048–1.556	0.016^*^
Treatment era	0.062			<0.001^*^	0.060			0.008^*^
2004 ~ 2008		Reference				Reference		
2009 ~ 2013		0.846	0.655–1.091	0.198		0.807	0.635–1.025	0.079
2014 ~ 2018		0.514	0.361–0.732	<0.001^*^		0.629	0.468–0.846	0.002^*^
Operative time ≥ 150 min	0.098	1.084	0.870–1.351	0.473	0.008	1.143	0.935–1.399	0.193
Stapled anastomosis	0.630				0.590			
Clinically significant AL	0.154	1.581	0.923–2.707	0.095	0.147	1.325	0.812–2.161	0.260
Histologic grade (PD)	<0.001	2.378	1.440–3.930	<0.001^*^	<0.001	2.229	1.402–3.544	<0.001^*^
pT stage ≥ 3	<0.001	1.422	1.094–1.849	0.008^*^	<0.001	1.708	1.332–2.191	<0.001^*^
pN stage ≥ 1	<0.001	1.706	1.367–2.129	<0.001^*^	<0.001	1.848	1.508–2.265	<0.001^*^
Lymphovascular invasion	<0.001	1.148	0.919–1.435	0.224	<0.001	1.123	0.916–1.379	0.265
Perineural invasion	<0.001	1.195	0.899–1.590	0.220	<0.001	1.370	1.068–1.757	0.013^*^
Harvested LN < 12	0.318				0.067	1.415	1.113–1.798	0.005^*^

^a^Variables with p < 0.20 in the univariate log-rank analysis were entered into the multivariable Cox regression model. Clinically significant anastomotic leakage and treatment era were retained in the multivariable model irrespective of their univariate significance, considering anastomotic leakage as the primary variable of interest and treatment era as a potential temporal confounder.

* Statistical significance, p < 0.05.

ASA, American Society of Anesthesiologist; BMI, body mass index; CI, confidence interval; HR, hazard ratio; LN, lymph node; AL, anastomotic leakage; PD, poorly differentiated

In the location-specific subgroup analyses, clinically significant AL was not independently associated with either OS or DFS in the colon cancer subgroup (OS: HR, 1.301; 95% CI, 0.532–3.182; p = 0.565; DFS: HR, 1.141; 95% CI, 0.468–2.782; p = 0.773) or the rectal cancer subgroup (OS: HR, 1.340; 95% CI, 0.661–2.718; p = 0.416; DFS: HR, 1.362; 95% CI, 0.741–2.502; p = 0.320) (S1 Table and S2 Table). In the colon cancer subgroup, older age, male sex, diabetes mellitus, chronic kidney disease, bowel obstruction, poorly differentiated histology, advanced pN stage, and treatment era were independently associated with OS, while BMI ≥ 25 kg/m² was associated with a lower risk of death. For DFS, older age, male sex, chronic kidney disease, poorly differentiated histology, advanced pT and pN stages, perineural invasion, and treatment era were independently associated with outcome, whereas BMI ≥ 25 kg/m² was associated with a lower risk of recurrence or death. In the rectal cancer subgroup, older age, diverting ileostomy, and advanced pT and pN stages were independently associated with worse OS. For DFS, older age, diverting ileostomy, poorly differentiated histology, and advanced pT and pN stages were independently associated with worse outcomes. NCRT and anastomotic level<5 cm were not independently associated with DFS after multivariable adjustment.

### Survival outcomes

The median follow-up duration was 59.3 months (range: 1.0 to 188.3 months). Kaplan-Meier survival analysis demonstrated no statistically significant differences in the 5-year OS (76.0% in the ALG vs. 84.1% in the NALG, p = 0.154) or the 5-year DFS (70.4% vs. 79.5%, p = 0.147). Survival curves are presented in Fig 2.

**Fig 2:**
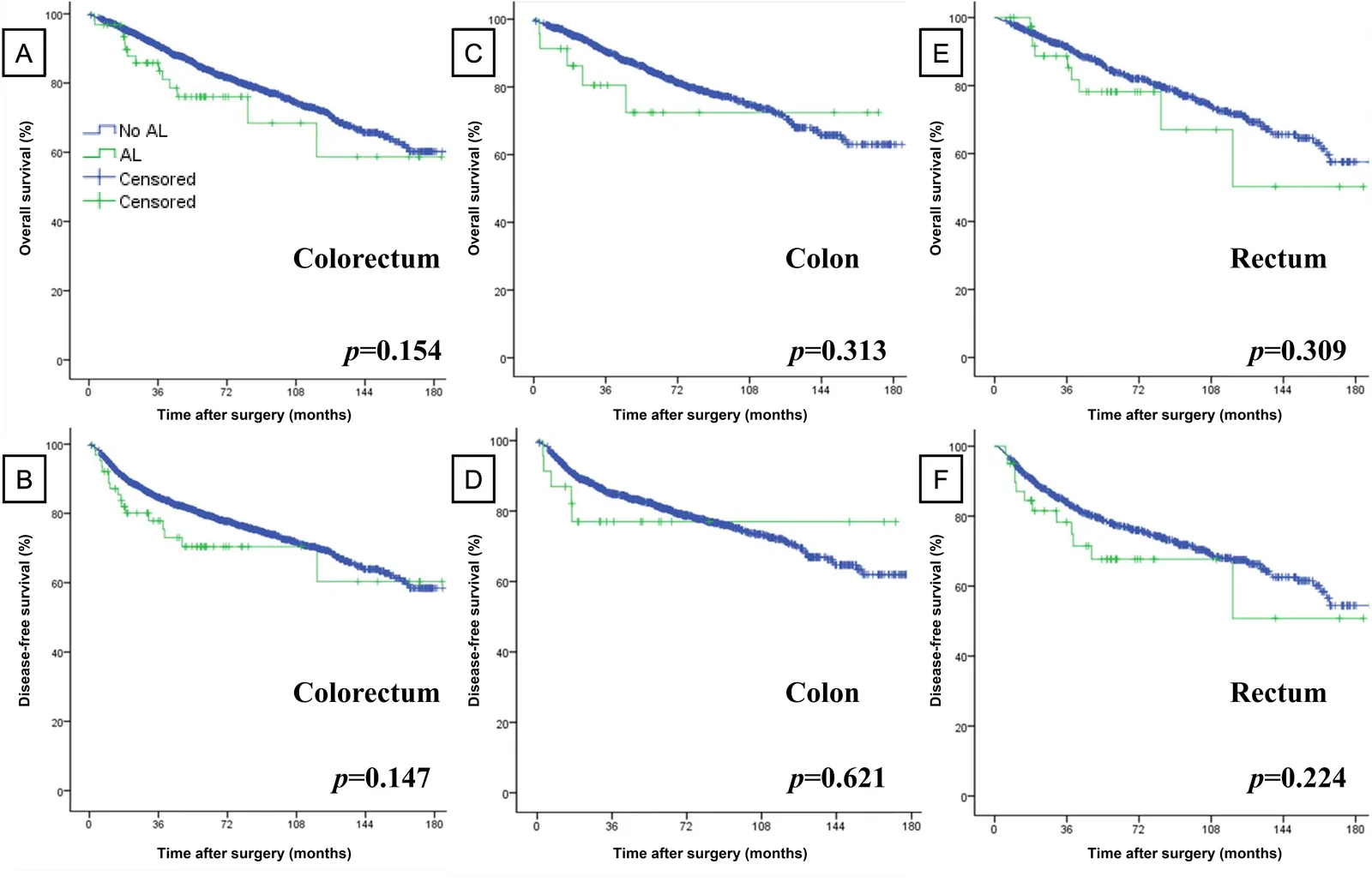
Kaplan-Meier analysis with log-rank test comparing survival in anastomotic leakage group (ALG) and no anastomotic leakage group (NALG) in relation to colon and rectal cancer. A. Overall survival (OS) for total patients, B. Disease-free survival (DFS) for total patients, C. OS for colon cancer, D. DFS for colon cancer, E. OS for rectal cancer, F. DFS for rectal cancer.

In subgroup analysis based on tumor location, patients with colon cancer showed no significant differences in the 5-year OS (72.4% in the ALG vs. 84.2% in the NALG, p = 0.313) or the 5-year DFS (77.0% vs. 80.8%, p = 0.621). Similarly, patients with rectal cancer showed no significant differences in the 5-year OS (78.2% in the ALG vs. 83.9% in the NALG, p = 0.309) or the 5-year DFS (67.7% vs. 77.5%, p = 0.224).

In subgroup analysis based on TNM staging revealed significantly worse 5-year OS and 5-year DFS in the ALG than in NALG for stage III colorectal cancer (56.3% vs. 76.3%, p = 0.005) and (43.9% vs. 68.4%, p = 0.007), respectively. In contrast, no significant differences in survival were observed between the two groups with stage I and II colorectal cancer (Fig 3). Furthermore, stage-specific subgroup analyses were performed separately for colon and rectal cancers. In the colon cancer group, there were no significant differences in 5-year OS or DFS between ALG and NALG for stage I (OS: p = 0.632; DFS: p = 0.607), stage II (OS: p = 0.410; DFS: p = 0.541), or stage III (OS: p = 0.276; DFS: p = 0.527) (Fig 4). In contrast, in the rectal cancer group, patients with stage III disease in the ALG showed significantly worse OS (p = 0.005) and DFS (p = 0.009) compared to those in the NALG, whereas no significant differences were observed in stage I (OS: p = 0.998; DFS: p = 0.398) or stage II (OS: p = 0.128; DFS: p = 0.074) (Fig 5).

**Fig 3:**
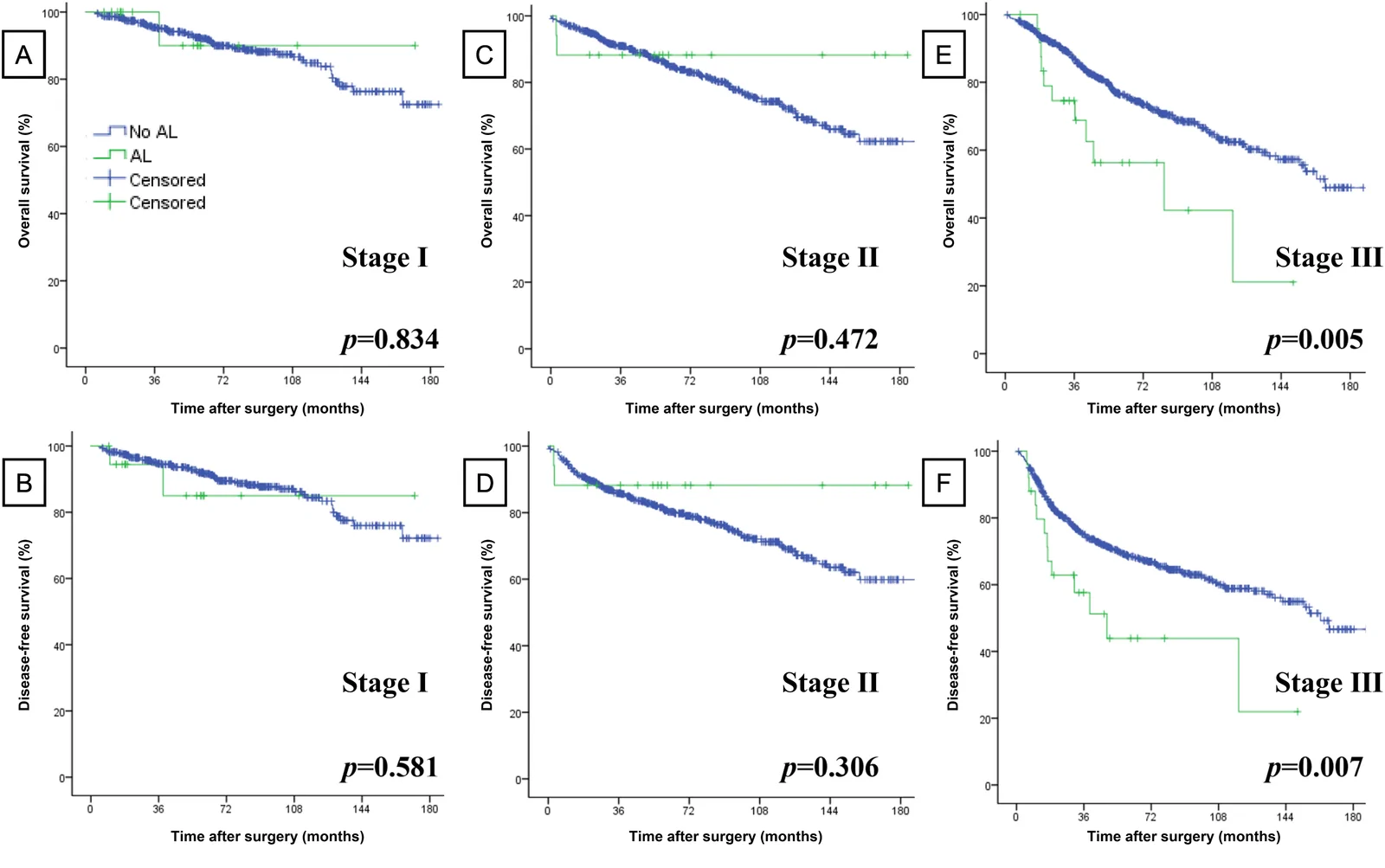
Kaplan-Meier analysis with log-rank test comparing survival in anastomotic leakage group (ALG) and no leakage group (NALG) in relation to TNM staging for colorectal cancer patients. A. Overall survival (OS) for stage Ⅰ patients, B. Disease-free survival (DFS) for stage Ⅰ patients, C. OS for stage Ⅱ patients, D. DFS for stage Ⅱ patients, E. OS for stage Ⅲ patients, F. DFS for stage Ⅲ patients.

**Fig 4:**
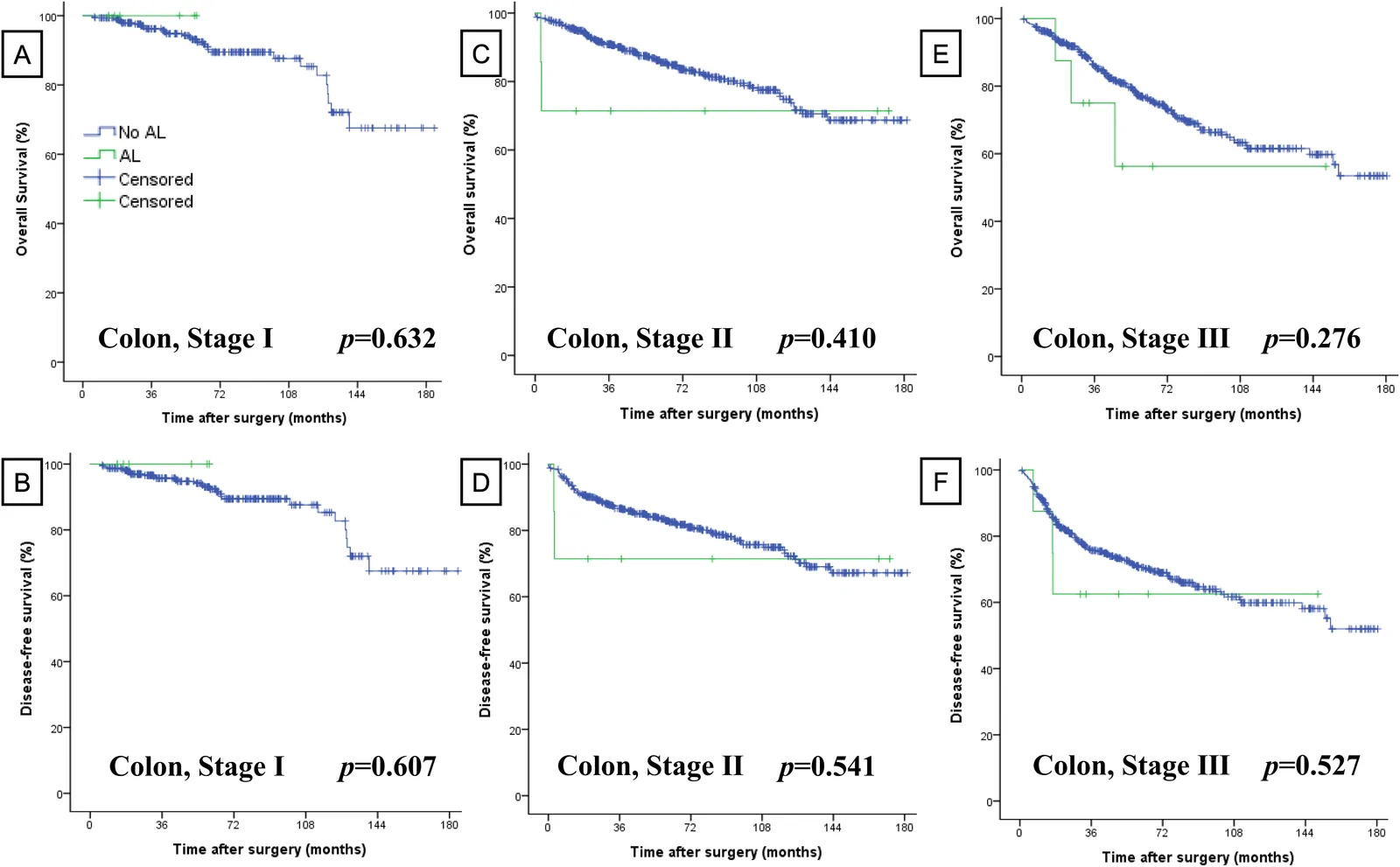
Kaplan-Meier analysis with log-rank test comparing survival in anastomotic leakage group (ALG) and no leakage group (NALG) in relation to TNM staging for colon cancer patients. A. Overall survival (OS) for stage Ⅰ patients, B. Disease-free survival (DFS) for stage Ⅰ patients, C. OS for stage Ⅱ patients, D. DFS for stage Ⅱ patients, E. OS for stage Ⅲ patients, F. DFS for stage Ⅲ patients.

**Fig 5:**
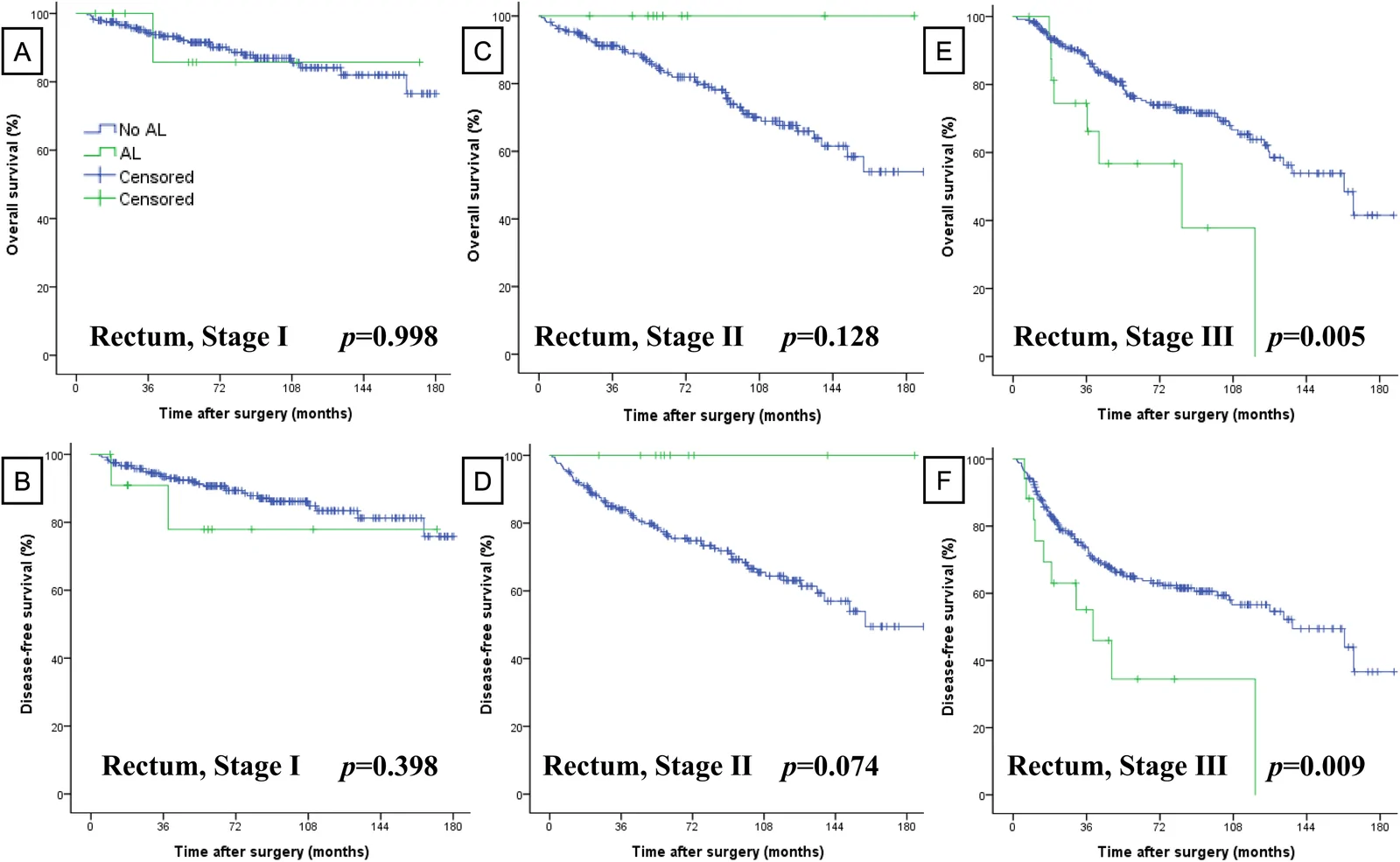
Kaplan-Meier analysis with log-rank test comparing survival in anastomotic leakage group (ALG) and no leakage group (NALG) in relation to TNM staging for rectal cancer patients. A. Overall survival (OS) for stage Ⅰ patients, B. Disease-free survival (DFS) for stage Ⅰ patients, C. OS for stage Ⅱ patients, D. DFS for stage Ⅱ patients, E. OS for stage Ⅲ patients, F. DFS for stage Ⅲ patients.

However, formal interaction analyses showed no significant interaction between clinically significant AL and tumor stage for either OS (p for interaction = 0.132) or DFS (p for interaction = 0.141) (Table 6). Likewise, the interaction between AL and tumor location was not significant for OS (p for interaction = 0.775) or DFS (p for interaction = 0.748). Although the adjusted association between AL and poorer survival was observed in the stage III subgroup (OS: HR, 2.36; 95% CI, 1.27–4.36; DFS: HR, 1.94; 95% CI, 1.10–3.41), the nonsignificant interaction tests did not provide statistical evidence that the association of AL with survival differed according to tumor stage or location.

**Table 6:** Interaction analyses between clinically significant anastomotic leakage and tumor stage or location for survival outcomes.

	Subgroup	Number of AL / Total number	Adjusted HR for AL (95% CI), OS	*p* for interaction	Adjusted HR for AL (95% CI), DFS	*p* for interaction
Tumor stage	Stage Ⅰ	19/575	0.92 (0.13–6.71)	0.132	1.29 (0.31–5.32)	0.141
	Stage Ⅱ	17/726	0.63 (0.16–2.57)		0.52 (0.13–2.10)	
	Stage Ⅲ	25/770	2.36 (1.27–4.36)		1.94 (1.10–3.41)	
Tumor location	Colon	23/1351	1.37 (0.56–3.34)	0.775	1.19 (0.49–2.89)	0.748
	Rectum	40/771	1.61 (0.82–3.18)		1.41 (0.78–2.54)	

### Adjuvant chemotherapy for Stage Ⅲ colorectal cancer

In stage Ⅲ colorectal cancer, rectal cancer was significantly more frequent in the ALG than in NALG (68.0% vs. 33.6%, p < 0.001). No significant differences regarding the administration of adjuvant chemotherapy were observed between the two groups (88.0% vs. 85.4%, p = 1.000). However, delay in the administration of adjuvant chemotherapy for more than two months after surgery was significantly more frequent in the ALG than in NALG (22.7% vs. 4.7%, p = 0.004) (Table 7).

**Table 7:** Adjuvant chemotherapy for Stage Ⅲ colorectal cancer.

Variables	ALG (n = 25), n (%)	NALG (n = 745), n (%)	p
Tumor location			<0.001^*^
Colon	8 (32.0)	495 (66.4)	
Rectum	17 (68.0)	250 (33.6)	
Adjuvant chemotherapy			1.000
Yes	22 (88.0)	636 (85.4)	
No	3 (12.0)	109 (14.6)	
Delay of adjuvant chemotherapy for more than 2 months^a^	5 (22.7)	30 (4.7)	0.004^*^

^a^Among patients who received chemotherapy.

* Statistical significance, p < 0.05.

## Discussion

In this study, we evaluated the long-term oncologic outcomes associated with AL in patients undergoing curative surgery for non-metastatic colorectal adenocarcinoma. Although AL has been consistently associated with increased short-term morbidity and mortality, its impact on long-term survival remains debatable. In our cohort of 2,122 patients, clinically significant AL was not independently associated with either OS or DFS after multivariable adjustment. In stage-stratified analyses, however, patients with stage III disease and AL showed worse OS and DFS, whereas no significant differences were observed in stages I and II. Importantly, formal interaction analyses did not demonstrate significant effect modification by tumor stage or location. Therefore, the stage- and location-specific findings should be interpreted as exploratory subgroup patterns rather than definitive evidence that the oncologic effect of AL differs according to disease stage or tumor location.

The different survival patterns observed across disease stages may have several possible explanations, although the absence of a significant AL-by-stage interaction precludes a definitive conclusion regarding stage-specific effect modification. Patients with early-stage colorectal cancer generally have a lower tumor burden and are less likely to require adjuvant chemotherapy, potentially reducing the clinical consequences of treatment delays following postoperative complications [23]. In contrast, patients with stage III disease have nodal involvement and routinely require postoperative systemic therapy. Thus, the inflammatory response associated with AL and delays in adjuvant chemotherapy could plausibly contribute to the poorer outcomes observed in the stage III subgroup. These mechanisms, however, remain hypothetical in the context of the present observational study. The inflammatory response triggered by AL may create a tumor-promoting microenvironment, as demonstrated in previous studies linking systemic inflammation to cancer recurrence and metastasis [24–26]. Moreover, AL often delays the initiation of adjuvant chemotherapy, a critical component of stage III colorectal cancer management [15,27–29]. Hershman et al. suggested that in stage III colon cancer, both all-cause mortality and cancer-specific survival were associated with an interval of more than two months between surgery and adjuvant chemotherapy [29]. Results from previous studies on the proportion of patients receiving adjuvant chemotherapy for stage III colorectal cancer in the group with AL compared to the group without AL, respectively, are as follows: 63% vs. 87%, p = 0.007 [27], 60.3% vs. 73.4%, p < 0.001 [15], and 37.5% vs. 88.1%, p = 0.004 [28]. In our study, although there were no statistical differences between the two groups in the proportion of patients receiving adjuvant chemotherapy (88.0% vs. 85.4%, p = 1.000), the proportion of patients experiencing a delay in administering adjuvant chemotherapy for more than 2 months was significantly higher in the ALG than in NALG (22.7% vs. 4.7%, p = 0.004). Our findings are consistent, to some extent, with those of previous studies reporting that delayed initiation of adjuvant chemotherapy is associated with adverse prognostic outcomes.

In the location- and stage-stratified analyses, no significant survival difference according to AL was observed at any stage of colon cancer, whereas worse OS and DFS were observed among patients with stage III rectal cancer. However, the formal interaction tests between AL and tumor location were not significant for either OS or DFS, and the interactions between AL and disease stage were also nonsignificant. Accordingly, these findings do not establish that the oncologic association of AL differs between colon and rectal cancer or across disease stages. The observed pattern in stage III rectal cancer should therefore be regarded as exploratory. Nevertheless, the higher incidence of AL in rectal cancer and the anatomical characteristics of the confined pelvic space provide biologically plausible reasons for further investigation of this subgroup [30].

Although the present findings do not support differential management of AL according to tumor stage or location, they reinforce the importance of prompt recognition and appropriate management of clinically significant AL after colorectal cancer surgery. In patients who require adjuvant chemotherapy, particularly those with stage III disease, postoperative recovery should be optimized to minimize unnecessary delays in systemic treatment. Enhanced recovery after surgery (ERAS) protocols, which prioritize early mobilization, nutritional support, and inflammation management, should be uniformly implemented to minimize the risk of AL and its complications [31,32]. A combination of an early warning score system and laboratory markers such as C-reactive protein, procalcitonin, and presepsin can facilitate the timely identification of AL [33–35].

This study is valuable due to the long follow-up period and comprehensive evaluation of the oncologic impact of AL, based on cancer stage and tumor location. However, the retrospective design introduces inherent selection and reporting biases. Furthermore, the study only included patients with clinically significant AL (grades B and C), potentially underestimating the full impact of AL on survival. AL grade A cases were excluded due to difficulty in identifying these cases in the retrospective review, as some records were missing. Given that grades B and C cases involved well-documented interventions or reoperations, the inclusion of grade A cases might have compromised the reliability of the database. Another limitation is the relatively small number of patients with AL, particularly after stratification by tumor location and disease stage. Although the overall study population was large, only 63 patients experienced clinically significant AL, resulting in small sample sizes in several subgroup analyses. This may have reduced statistical power, increased the risk of type II error. Finally, the 15-year study period encompassed substantial changes in surgical techniques, perioperative management, neoadjuvant treatment, and systemic chemotherapy. Although treatment era was incorporated into the multivariable models to account for temporal changes in clinical practice, categorization by calendar period cannot fully capture changes in individual treatment regimens or perioperative strategies, and residual temporal confounding may remain. In addition, despite adjustment for several newly incorporated comorbidities and clinical variables, residual confounding from unmeasured or incompletely measured factors cannot be excluded.

Further prospective multicenter studies are needed to validate our findings and to explore the association between AL and oncologic outcomes. Investigating the significance of systemic inflammation and immune response biomarkers could provide deeper insights into the role of AL in tumor progression. Additionally, standardized perioperative care protocols should be implemented to optimize the outcomes for high-risk patients, particularly those with advanced-stage disease.

## Conclusions

In conclusion, clinically significant AL was not independently associated with long-term oncologic outcomes after curative colorectal cancer surgery. Although poorer survival was observed in patients with stage III disease, particularly in the rectal cancer subgroup, formal interaction analyses did not demonstrate significant effect modification by tumor stage or location. These subgroup findings should therefore be interpreted cautiously and require validation in larger multicenter cohorts. Prompt management of AL and avoidance of unnecessary delays in adjuvant chemotherapy remain clinically important, particularly for patients requiring postoperative systemic treatment.

## Supporting information

S1 Table Univariate and multivariate analyses for overall and disease-free survival in colon cancer. (DOCX)

S2 Table Univariate and multivariate analyses for overall and disease-free survival in rectal cancer. (DOCX)

S1 Dataset De-identified dataset used for the analyses in this study. (XLSX)
