# Supplementary material for: Long-term oncologic outcomes associated with clinically significant anastomotic leakage after curative colorectal cancer surgery: Stage- and location-stratified analyses
Source: PLoS One. 2026 Oct 1;21(10):e0359416. doi: 10.1371/journal.pone.0359416 (PMC13630224; doi:10.1371/journal.pone.0359416)
Supplement: Supplementary file 2 [file pone.0359416.s002.docx]

S2 Table. Univariate and multivariate analyses for overall and disease-free survival in rectal cancer.

| Variables |  | Overall survival | | | | |  | Disease-free survival | | | | |
| --- | --- | --- | --- | --- | --- | --- | --- | --- | --- | --- | --- | --- |
|  |  | Univariate log-rank test |  | Multivariate Cox regression analysis^a^ | | |  | Univariate log-rank test |  | Multivariate Cox regression analysis^a^ | | |
|  |  | *p* |  | HR | 95% CI | *p* |  | *p* |  | HR | 95% CI | *p* |
| Age ≥ 70 years |  | <0.001 |  | 2.705 | 1.943–3.766 | <0.001^*^ |  | <0.001 |  | 2.133 | 1.582–2.876 | <0.001^*^ |
| Male sex |  | 0.042 |  | 1.367 | 0.972–1.924 | 0.073 |  | 0.080 |  | 1.220 | 0.897–1.660 | 0.205 |
| BMI ≥ 25 kg/m^2^ |  | 0.972 |  |  |  |  |  | 0.876 |  |  |  |  |
| ASA > 1 |  | 0.046 |  | 1.397 | 0.737–2.648 | 0.306 |  | 0.101 |  | 1.260 | 0.744–2.136 | 0.390 |
| Cardiovascular disease |  | 0.163 |  | 1.082 | 0.772–1.517 | 0.647 |  | 0.206 |  |  |  |  |
| Diabetes Mellitus |  | 0.663 |  |  |  |  |  | 0.515 |  |  |  |  |
| Chronic kidney disease |  | 0.837 |  |  |  |  |  | 0.899 |  |  |  |  |
| Serum albumin<3.5, g/dL |  | 0.560 |  |  |  |  |  | 0.358 |  |  |  |  |
| Bowel obstruction |  | 0.064 |  | 2.132 | 0.835–5.446 | 0.114 |  | 0.390 |  |  |  |  |
| Treatment era |  | 0.627 |  |  |  | 0.124^a^ |  | 0.827 |  |  |  | 0.309 |
| 2004~2008 |  |  |  | Reference |  |  |  |  |  | Reference |  |  |
| 2009~2013 |  |  |  | 1.151 | 0.778–1.704 | 0.481 |  |  |  | 1.027 | 0.720–1.466 | 0.883 |
| 2014~2018 |  |  |  | 0.678 | 0.386–1.192 | 0.177 |  |  |  | 0.761 | 0.492–1.176 | 0.218 |
| NCRT |  | 0.299 |  |  |  |  |  | 0.096 |  | 1.182 | 0.816–1.713 | 0.377 |
| Diverting ileostomy |  | 0.006 |  | 1.797 | 1.129–2.862 | 0.014^*^ |  | 0.014 |  | 1.574 | 1.010–2.453 | 0.045^*^ |
| Anastomotic level from anal verge < 5 cm |  | 0.024 |  | 0.995 | 0.623–1.590 | 0.985 |  | 0.044 |  | 0.948 | 0.631–1.425 | 0.798 |
| Operative time ≥ 150 min |  | 0.245 |  |  |  |  |  | 0.289 |  |  |  |  |
| Stapled anastomosis |  | 0.589 |  |  |  |  |  | 0.257 |  |  |  |  |
| Clinically significant AL |  | 0.309 |  | 1.340 | 0.661–2.718 | 0.416 |  | 0.224 |  | 1.362 | 0.741–2.502 | 0.320 |
| Histologic grade (PD) |  | 0.789 |  |  |  |  |  | 0.008 |  | 3.588 | 1.070–12.029 | 0.038^*^ |
| pT stage ≥ 3 |  | <0.001 |  | 1.863 | 1.286–2.699 | 0.001^*^ |  | <0.001 |  | 1.795 | 1.280–2.517 | 0.001^*^ |
| pN stage ≥ 1 |  | <0.001 |  | 1.545 | 1.087–2.194 | 0.015^*^ |  | <0.001 |  | 1.804 | 1.320–2.466 | <0.001^*^ |
| Lymphovascular invasion |  | 0.001 |  | 1.203 | 0.837–1.728 | 0.319 |  | <0.001 |  | 1.231 | 0.888–1.707 | 0.213 |
| Perineural invasion |  | 0.015 |  | 1.087 | 0.656–1.800 | 0.746 |  | <0.001 |  | 1.263 | 0.827–1.928 | 0.279 |
| Harvested LN < 12 |  | 0.608 |  |  |  |  |  | 0.211 |  |  |  |  |

^a^Variables with p <0.20 in the univariate log-rank analysis were entered into the multivariable Cox regression model. Clinically significant anastomotic leakage and treatment era were retained in the multivariable model irrespective of their univariate significance, considering anastomotic leakage as the primary variable of interest and treatment era as a potential temporal confounder.

^*^Statistical significance, p < 0.05.

ASA, American Society of Anesthesiologist; BMI, body mass index; NCRT, neoadjuvant chemoradiotherapy; CI, confidence interval; HR, hazard ratio; LN, lymph node; AL, anastomotic leakage; PD, poorly differentiated
