# Supplementary material for: Long-term oncologic outcomes associated with clinically significant anastomotic leakage after curative colorectal cancer surgery: Stage- and location-stratified analyses
Source: PLoS One. 2026 Oct 1;21(10):e0359416. doi: 10.1371/journal.pone.0359416 (PMC13630224; doi:10.1371/journal.pone.0359416)
Supplement: Supplementary file 3 [file pone.0359416.s005.docx]

Response to Reviewers (PLOS ONE)

**● Reviewer #1:**

This study aimed to evaluate the long-term oncologic impact of anastomotic leakage, with particular focus on stage- and location-specific differences, in patients undergoing curative colorectal cancer surgery. The authors reported stage- and location-specific differences, with a potential adverse impact observed in patients with stage III rectal cancer.

However, several important potential confounding factors, such as nutritional status and comorbidities, were not assessed in the analysis. These variables may substantially influence both postoperative complications and long-term oncologic outcomes. Therefore, I would suggest reanalyzing the data after incorporating these important confounders to improve the validity and interpretability of the findings.

**Answer)**

Thank you for this important comment. In the original analysis, ASA classification and BMI were included as general indicators of comorbidity and nutritional status. However, we agree that these measures alone may not adequately capture the potential confounding effects of underlying comorbidities and nutritional impairment. Therefore, we additionally evaluated cardiovascular disease, diabetes mellitus, chronic kidney disease, and preoperative hypoalbuminemia, defined as a serum albumin level of <3.5 g/dL within 30 days before surgery. These variables were assessed in the univariate survival analyses and were entered into the multivariable Cox regression models when the predefined criterion of p<0.20 was met. Clinically significant AL and treatment era were retained irrespective of their univariate significance.

After incorporating these additional variables and treatment era, clinically significant AL remained not independently associated with either OS (HR, 1.581; 95% CI, 0.923–2.707; p=0.095) or DFS (HR, 1.325; 95% CI, 0.812–2.161; p=0.260). The Methods, Results, Table 2, Table 5, and the location-specific supplementary analyses have been revised accordingly.

**● Reviewer #2:**

Thank you for the opportunity to review this retrospective single-centre study of 2,122 patients, assessing whether clinically significant anastomotic leakage (Grade B/C) affects long-term oncologic outcomes after curative colorectal cancer surgery. Overall, AL was not an independent predictor of OS or DFS on multivariable analysis. However, subgroup analyses suggested worse outcomes in stage III disease, particularly stage III rectal cancer, potentially related to delays in adjuvant chemotherapy. My comments are as follows:

1. The primary analysis was negative (AL not independently associated with OS or DFS). The positive findings are based on small subgroup analyses of only 63 leak patients and should be over-relied on. The manuscript would be strengthened by presenting these findings more cautiously and emphasising that the primary multivariable analysis was negative.

**Answer)**

We agree that the primary multivariable analyses did not demonstrate an independent association between clinically significant AL and either OS or DFS. In the revised analyses incorporating additional clinical variables and treatment era, AL remained not independently associated with OS (HR, 1.581; 95% CI, 0.923–2.707; p=0.095) or DFS (HR, 1.325; 95% CI, 0.812–2.161; p=0.260).

We therefore revised the Abstract, Results, Discussion, and Conclusions to emphasize the negative primary multivariable findings and to present the stage- and location-specific findings more cautiously. In addition, formal interaction analyses were performed, as suggested in Comment 2 below. Because these interaction tests did not demonstrate significant effect modification by either tumor stage or location, the subgroup findings are now described as exploratory observations rather than definitive evidence of differential oncologic effects. We have also emphasized the limited number of AL events and the resulting reduced statistical power of subgroup analyses in the Limitations section.

2. The manuscript's central conclusion is based on subgroup analyses stratified by stage and tumour location. However, formal interaction testing was not performed. Demonstration of statistical significance in one subgroup and non-significance in another does not establish a differential effect. The authors should perform interaction analyses (AL × stage and AL × tumour location) before concluding that the oncologic impact of AL differs according to disease stage or tumour location.
**Answer)**

Thank you for the important comment. We agree with the reviewer that statistical significance in one subgroup and non-significance in another does not, by itself, establish a differential effect. Accordingly, we performed formal interaction analyses using multivariable Cox proportional hazards models to evaluate whether the association between clinically significant AL and survival outcomes differed according to tumor stage or tumor location. Interaction terms between AL and tumor stage (AL × stage) and between AL and tumor location (AL × location) were evaluated separately for OS and DFS. Stage 0 was excluded from the stage interaction analysis because of its small sample size and clinical heterogeneity.

The interactions between AL and tumor stage were not statistically significant for either OS (p for interaction=0.132) or DFS (p for interaction=0.141). Likewise, there was no significant interaction between AL and tumor location for OS (p for interaction=0.775) or DFS (p for interaction=0.748). Although the stage III subgroup showed a significant adjusted association between AL and poorer OS (HR, 2.36; 95% CI, 1.27–4.36) and DFS (HR, 1.94; 95% CI, 1.10–3.41), the nonsignificant interaction tests indicate that these findings do not provide statistical evidence that the effect of AL differs according to tumor stage or location.

We have added the interaction analyses to the Methods and Results and present the findings in a new Table 6. The Abstract, Discussion, and Conclusions have also been revised so that the stage- and location-specific findings are interpreted as exploratory rather than as evidence of established effect modification.

3. AL patients had significantly more rectal cancers. Residual confounding may remain despite adjustment, and methods such as propensity matching could strengthen the analysis. The authors should consider reporting separate leak rates for colon and rectal cancer.
**Answer)**

We agree that the higher proportion of rectal cancer in the AL group could contribute to confounding. We have now explicitly reported the incidence of clinically significant AL according to tumor location: AL occurred in 23 of 1,351 patients with colon cancer (1.7%) and in 40 of 771 patients with rectal cancer (5.2%).

Tumor location was evaluated as a potential prognostic variable in the overall survival analyses and was entered into the multivariable model when it met the predefined univariate selection criterion. In addition, we performed separate multivariable Cox regression analyses for colon and rectal cancer and formal interaction analyses between AL and tumor location. The AL-by-location interaction was not significant for either OS (p for interaction=0.775) or DFS (p for interaction=0.748).

We also considered propensity score matching as suggested by the reviewer. However, because only 63 patients experienced clinically significant AL, propensity score matching would substantially reduce the effective sample size and statistical precision, particularly for the stage- and location-specific analyses. We therefore retained the full-cohort multivariable Cox regression as the primary approach and supplemented it with location-specific Cox regression and formal interaction analyses. The possibility of residual confounding from unmeasured factors has also been emphasized in the Limitations section.

4. The 2004–2018 timeframe spans major changes in surgery, perioperative care, neoadjuvant treatment and chemotherapy. Treatment era should be considered as a potential confounder.
**Answer)**

We agree that the long study period may have introduced temporal heterogeneity related to changes in surgical techniques, perioperative management, neoadjuvant treatment, and systemic chemotherapy. To address this potential confounding, we categorized the study period into three 5-year treatment eras (2004–2008, 2009–2013, and 2014–2018) according to the year of surgery and incorporated treatment era as an additional covariate in the revised multivariable Cox regression analyses.

After adjustment for treatment era, the association between anastomotic leakage and long-term oncologic outcomes remained essentially unchanged, and AL was not independently associated with either overall survival or disease-free survival. We have added the treatment-era variable to the Methods, Results, and relevant tables. We have also acknowledged in the Discussion that treatment practices evolved over the 15-year study period and that adjustment for calendar era may not fully reflect all changes in clinical practice.

5. The study evaluates clinically significant AL rather than all AL (grade A leaks excluded). This distinction should be stated consistently throughout the manuscript.

**Answer)**

We agree that our study specifically evaluated clinically significant AL rather than all grades of AL. In this study, AL was defined as grade B or C leakage according to the International Study Group of Rectal Cancer (ISREC) classification, whereas grade A leakage, which does not require active therapeutic intervention, was excluded.

Accordingly, we have revised the manuscript to consistently clarify that the AL group represents patients with ‘clinically significant anastomotic leakage (grade B or C)’. This terminology has been clarified in the overall manuscript and title.

**● Reviewer #3:**

This is an interesting paper on the effects of anastomotic leak for colorectal cancer patients.

I have some points that I think should be addressed:

Introduction

Page 5 lines 6-8
Have there been any meta-analyses to support the points made here? If yes, they should be included.
**Answer)**

We have added recent systematic reviews and meta-analyses to provide stronger evidence regarding the incidence of anastomotic leakage after colorectal cancer surgery. We have revised the Introduction accordingly and added the relevant references.

Line 15
Please cite papers for the limited data on colon cancer
**Answer)**

We agree that the statement regarding the relatively limited evidence for colon cancer should be supported by appropriate references. We have therefore added references to previous studies specifically evaluating the oncologic impact of anastomotic leakage after colon cancer surgery. We also revised the sentence to avoid overstating the paucity of available evidence, as follows:

“Previous studies on the long-term oncologic impact of AL have predominantly focused on rectal cancer, whereas relatively fewer studies have specifically evaluated patients with colon cancer.”

Methods
Page 7
A reference for the AJCC 7th edition should be included.
**Answer)**

We added a reference for the AJCC 7^th^ edition.

What was the definition of Stage 0 found in Table 4?
**Answer)**

Thank you for this important comment. In the present study, Stage 0 comprised patients with pTisN0M0 disease after upfront surgery and those with ypT0N0M0 disease after NCRT. We have clarified this definition in the footnote of Table 4.

Furthermore, patients with stage 0 disease were included in the overall cohort because they met the study eligibility criteria. However, they were not included in the stage-specific survival analyses, which focused on stages I–III. This was because the number of patients with stage 0 disease was small (n=51, 2.4% of the entire cohort), with only two patients experiencing clinically significant anastomotic leakage, precluding a meaningful and statistically reliable stage-specific comparison. We have added this explanation to the methods section.

Results
Page 8
The number of colon and rectal patients should be stated, and the breakdown of how many colon or rectal patients had grade B or C AL
**Answer)**

We have revised the results section to provide the number of patients according to tumor location and the distribution of grade B and C AL according to tumor location. Among the 2,122 patients, 1,351 had colon cancer and 771 had rectal cancer. Of the 63 patients who developed clinically significant AL, 23 patients had colon cancer and 40 patients had rectal cancer. Among patients with colon cancer, 7 patients had grade B and 16 patients had grade C AL, whereas among those with rectal cancer, 11 patients had grade B and 29 patients had grade C AL. These data have been added to the results section.

Why was 70 chosen as the dividing point for age? This should be explained
**Answer)**

The cutoff of 70 years was selected primarily based on our institutional practice for preoperative risk assessment. At our institution, patients aged 70 years or older routinely undergo additional preoperative evaluation by both cardiology and pulmonology, regardless of the presence of underlying cardiopulmonary comorbidities. Therefore, age was categorized as <70 and ≥70 years to reflect this clinically relevant institutional criterion rather than on the basis of a data-driven cutoff. In addition, an age threshold of approximately 70 years has also been commonly used in previous studies and recommendations addressing older patients with colorectal cancer. We have clarified the rationale for this age cutoff in the methods section and added relevant reference.

Why was 25 and over chosen for BMI? This should be explained, and whether the authors are using the WHO BMI guidelines for Asian patients
**Answer)**

BMI was categorized as <25 and ≥25 kg/m², with BMI ≥25 kg/m² defined as obesity according to the Korean Society for the Study of Obesity guidelines. We have clarified the rationale for this BMI cutoff in the methods section and added relevant reference.

**Additional revision:**

While revising the manuscript, we also reviewed the cutoff used for operative time. Because the previous cutoff of 240 minutes lacked a clearly established clinical basis, operative time was reclassified using the cohort median of 150 minutes, and all relevant analyses were repeated. The Methods and relevant tables have been updated accordingly.

How were rectal cancer patients who had neoadjuvant chemoradiotherapy considered in the results?
**Answer)**

Thank you for this important comment. Rectal cancer patients who received neoadjuvant chemoradiotherapy (NCRT) were included in both the overall cohort and the rectal cancer subgroup analyses. For stage-specific analyses, postoperative pathologic stage was used; pTNM was applied to patients undergoing upfront surgery, whereas ypTNM was applied to patients receiving NCRT.

NCRT was also evaluated as a clinicopathologic variable in the rectal cancer-specific survival analyses. According to our predefined variable-selection criterion, NCRT was not entered into the multivariable OS model because its univariate p value was ≥0.20, whereas it was entered into the multivariable DFS model (univariate p=0.096) and was not independently associated with DFS after adjustment (HR, 1.182; 95% CI, 0.816–1.713; p=0.377).

We additionally reported the proportion of patients receiving NCRT in Table 2. Because NCRT was applicable only to patients with rectal cancer, the corresponding numbers and percentages were calculated among rectal cancer patients only. NCRT was not included as a covariate in the Cox regression analysis of the entire colorectal cancer cohort because its applicability was inherently dependent on tumor location.

Page 10
I think supplemental information and analyses for colon and rectal separately would be useful for readers.
**Answer)**

Separate subgroup analyses for colon and rectal cancer are presented in Supplementary Tables 1 and 2 (newly S1 Table, and S2 Table), respectively. These analyses include univariate log-rank and multivariable Cox regression analyses for OS and DFS. In the rectal cancer subgroup, rectal cancer-specific variables, including NCRT, diverting ileostomy, and anastomotic level from the anal verge (<5 cm vs. ≥5 cm), were additionally evaluated.

In the revised analyses, clinically significant AL was not independently associated with either OS or DFS in the colon cancer subgroup (OS: HR, 1.301; 95% CI, 0.532–3.182; p=0.565; DFS: HR, 1.141; 95% CI, 0.468–2.782; p=0.773) or in the rectal cancer subgroup (OS: HR, 1.340; 95% CI, 0.661–2.718; p=0.416; DFS: HR, 1.362; 95% CI, 0.741–2.502; p=0.320). The Results section has been expanded to summarize these location-specific findings and the significant independent prognostic factors identified in each subgroup.

Discussion/Conclusions
Given that patients with AL having issues after surgery for CRC is not new. I think a paragraph should be added explaining what this paper contributes to the literature and why it is important.
**Answer)**

We agree that the adverse short-term consequences of AL after colorectal cancer surgery are already well established. The contribution of the present study is therefore not simply to demonstrate that AL is harmful, but to provide a comprehensive evaluation of its association with long-term oncologic outcomes within a single colorectal cancer cohort, incorporating overall multivariable analyses, stage- and location-stratified analyses, and formal interaction testing.

Our revised analyses showed that clinically significant AL was not independently associated with OS or DFS in the overall cohort. Although poorer survival was observed among patients with stage III disease, particularly in the rectal cancer subgroup, formal interaction analyses did not demonstrate significant effect modification by either tumor stage or location. Thus, our findings provide a more cautious interpretation of the apparent subgroup patterns and demonstrate that statistical differences observed within individual subgroups should not be considered evidence of a differential AL effect without formal interaction testing.

In addition, the study evaluates both colon and rectal cancer within a consistent analytic framework and examines clinically relevant factors that may contribute to poorer outcomes after AL, including delays in the initiation of adjuvant chemotherapy among patients with stage III disease. We have revised the Discussion and Conclusions to clarify these contributions while explicitly acknowledging the exploratory nature of the subgroup findings.

Limitations section
The low number of patients in some groups in the analyses is not included and should be.
**Answer)**

We have expanded the Limitations section to address this issue. Although the overall study population was large, only 63 patients experienced clinically significant AL, and the number of AL events became considerably smaller after stratification by tumor stage and location. This may have reduced statistical power, widened the confidence intervals of subgroup estimates, and increased the risk of type II error, including limited power to detect interaction effects. Therefore, the stage- and location-specific subgroup findings are now explicitly described as exploratory and requiring validation in larger multicenter cohorts.

Tables 2, 3, 4
What statistical analyses were done to create these p-values? This would be useful in the text and on the Table legends
**Answer)**

We have clarified the statistical methods used for comparisons in Tables 2–4. Categorical variables were compared using Pearson’s chi-square test or Fisher’s exact test, as appropriate according to the expected cell counts. This information has been added to the statistical analysis section of the methods and to the legends of Tables 2–4.

Table 4
WD/MD and PD are not defined
**Answer)**

We have clarified the definitions of WD/MD and PD directly in Table 4. WD/MD denotes well/moderately differentiated tumors, and PD denotes poorly differentiated tumors. The table and its footnote have been revised accordingly.

General points
There are only 25 references in the paper, and none from 2025 or 2026. I recommend a search on PubMed for AL and CRC or AL and colon cancer, as I found numerous papers looking at AL and colon/CRC and postoperative mortality and morbidity.
**Answer)**

We performed an updated PubMed search using terms including “anastomotic leakage,” “colorectal cancer,” “colon cancer,” “mortality,” and “morbidity,” with particular attention to studies published in 2025 and 2026. Based on this updated search, we added recent publications relevant to the definition, reporting, and clinical consequences of AL, including the 2025 CoReAL reporting framework by Heuvelings et al., the 2025 study by Gosavi et al. evaluating morbidity and non-oncologic mortality after AL following colon cancer resection, and the 2026 study by Müller et al. evaluating long-term survival after AL in colon cancer. The Introduction and Discussion have been updated accordingly.

I would be interested in the author's thoughts on whether this paper would be better as two separate papers on colon cancer and rectal cancer.

**Answer)**

Thank you for this thoughtful comment. We agree that colon and rectal cancers differ in several important clinical and treatment-related aspects, and we carefully considered whether they should be evaluated separately.

However, we believe that retaining both tumor locations within a single study is particularly appropriate for the present research question because it enables direct comparison of the association between AL and outcomes according to tumor location within the same cohort and analytic framework. Importantly, the revised study now includes a formal AL-by-tumor-location interaction analysis, which directly evaluates whether the association of AL with survival differs between colon and rectal cancer. Such a comparison would not be possible in the same manner if the cohort were divided into two independent studies.

In addition, the number of patients with clinically significant AL was relatively small, particularly after stratification (23 patients with colon cancer and 40 with rectal cancer). Dividing the cohort into two independent studies would further reduce the statistical power of each analysis, particularly for stage-specific comparisons.

For these reasons, we believe that presenting colon and rectal cancer together, while providing detailed location-specific analyses, allows a more informative assessment of the association between AL and oncologic outcomes than separating the data into two individual papers.

**Thank you again for your insightful comments and suggestions. We sincerely appreciate the time and effort you devoted to reviewing our manuscript and providing constructive comments.**

**Sincerely,**

**Jeong-Heum Baek, M.D., Ph.D.**

**(On behalf of all co-authors)**
