# Supplementary material for: Long-term oncologic outcomes associated with clinically significant anastomotic leakage after curative colorectal cancer surgery: Stage- and location-stratified analyses
Source: PLoS One. 2026 Oct 1;21(10):e0359416. doi: 10.1371/journal.pone.0359416 (PMC13630224; doi:10.1371/journal.pone.0359416)
Supplement: Peer review history

# Peer review: Decision Letter 0

**DOI:** 10.1371/journal.pone.0359416.r001

**Authors:** Chong-Chi Chiu

19 Jun 2026

Dear Dr. Baek,

Thank you for submitting your manuscript to PLOS ONE. After careful consideration, we feel that it has merit but does not fully meet PLOS ONE’s publication criteria as it currently stands. Therefore, we invite you to submit a revised version of the manuscript that addresses the points raised during the review process.

Please submit your revised manuscript by Aug 18 2026 11:59PM. If you will need more time than this to complete your revisions, please reply to this message or contact the journal office at plosone@plos.org. When you're ready to submit your revision, log on to https://www.editorialmanager.com/pone/ and select the 'Submissions Needing Revision' folder to locate your manuscript file.

- A letter that responds to each point raised by the academic editor and reviewer(s). You should upload this letter as a separate file labeled 'Response to Reviewers'.
- A marked-up copy of your manuscript that highlights changes made to the original version. You should upload this as a separate file labeled 'Revised Manuscript with Track Changes'.
- An unmarked version of your revised paper without tracked changes. You should upload this as a separate file labeled 'Manuscript'.

If you would like to make changes to your financial disclosure, please include your updated statement in your cover letter. Guidelines for resubmitting your figure files are available below the reviewer comments at the end of this letter.

If applicable, we recommend that you deposit your laboratory protocols in protocols.io to enhance the reproducibility of your results. Protocols.io assigns your protocol its own identifier (DOI) so that it can be cited independently in the future. For instructions see: https://journals.plos.org/plosone/s/submission-guidelines#loc-laboratory-protocols. Additionally, PLOS ONE offers an option for publishing peer-reviewed Lab Protocol articles, which describe protocols hosted on protocols.io. Read more information on sharing protocols at https://plos.org/protocols?utm_medium=editorial-email&utm_source=authorletters&utm_campaign=protocols.

As the corresponding author, your ORCID iD is verified in the submission system and will appear in the published article. PLOS supports the use of ORCID, and we encourage all coauthors to register for an ORCID iD and use it as well. Please encourage your coauthors to verify their ORCID iD within the submission system before final acceptance, as unverified ORCID iDs will not appear in the published article. *Only*  the individual author can complete the verification step; PLOS staff *cannot*  verify ORCID iDs on behalf of authors.

We look forward to receiving your revised manuscript.

Kind regards,

Chong-Chi Chiu

Academic Editor

PLOS One

Journal Requirements:

When submitting your revision, we need you to address these additional requirements.

1. Please ensure that your manuscript meets PLOS ONE's style requirements, including those for file naming. The PLOS ONE style templates can be found at

https://journals.plos.org/plosone/s/file?id=wjVg/PLOSOne_formatting_sample_main_body.pdf and https://journals.plos.org/plosone/s/file?id=ba62/PLOSOne_formatting_sample_title_authors_affiliations.pdf

2. Thank you for stating the following financial disclosure:

This work was supported by the Gachon University Research Fund of 2023 (GCU-202308190001).

Please state what role the funders took in the study. If the funders had no role, please state: "The funders had no role in study design, data collection and analysis, decision to publish, or preparation of the manuscript."

If this statement is not correct you must amend it as needed.

Please include this amended Role of Funder statement in your cover letter; we will change the online submission form on your behalf.

3. Please include captions for your Supporting Information files at the end of your manuscript, and update any in-text citations to match accordingly. Please see our Supporting Information guidelines for more information: http://journals.plos.org/plosone/s/supporting-information.

4. We note that there is identifying data in the Supporting Information file <Anastomotic leakage oncologic outcomes_DB_PLOS One.xlsx>. Due to the inclusion of these potentially identifying data, we have removed this file from your file inventory. Prior to sharing human research participant data, authors should consult with an ethics committee to ensure data are shared in accordance with participant consent and all applicable local laws.

Data sharing should never compromise participant privacy. It is therefore not appropriate to publicly share personally identifiable data on human research participants. The following are examples of data that should not be shared:

-Name, initials, physical address

-Ages more specific than whole numbers

-Internet protocol (IP) address

-Specific dates (birth dates, death dates, examination dates, etc.)

-Contact information such as phone number or email address

-Location data

-ID numbers that seem specific (long numbers, include initials, titled “Hospital ID”) rather than random (small numbers in numerical order)

Data that are not directly identifying may also be inappropriate to share, as in combination they can become identifying. For example, data collected from a small group of participants, vulnerable populations, or private groups should not be shared if they involve indirect identifiers (such as sex, ethnicity, location, etc.) that may risk the identification of study participants.

Additional guidance on preparing raw data for publication can be found in our Data Policy (https://journals.plos.org/plosone/s/data-availability#loc-human-research-participant-data-and-other-sensitive-data) and in the following article: http://www.bmj.com/content/340/bmj.c181.long.

Please remove or anonymize all personal information (<specific identifying information in file to be removed>), ensure that the data shared are in accordance with participant consent, and re-upload a fully anonymized data set. Please note that spreadsheet columns with personal information must be removed and not hidden as all hidden columns will appear in the published file.

5. If the reviewer comments include a recommendation to cite specific previously published works, please review and evaluate these publications to determine whether they are relevant and should be cited. There is no requirement to cite these works unless the editor has indicated otherwise.

Additional Editor Comments :

The reviewers have completed their assessment of your manuscript. Please revise the manuscript carefully in accordance with their comments and recommendations.

Reviewers' comments:

Reviewer's Responses to Questions

**Comments to the Author**

1. Is the manuscript technically sound, and do the data support the conclusions?

Reviewer #1: Yes

Reviewer #2: Partly

Reviewer #3: Yes

**********

2. Has the statistical analysis been performed appropriately and rigorously?

Reviewer #1: Yes

Reviewer #2: Yes

Reviewer #3: Yes

**********

3. Have the authors made all data underlying the findings in their manuscript fully available?

The PLOS Data policy

Reviewer #1: Yes

Reviewer #2: Yes

Reviewer #3: Yes

**********

4. Is the manuscript presented in an intelligible fashion and written in standard English?

Reviewer #1: Yes

Reviewer #2: Yes

Reviewer #3: Yes

**********

Reviewer #1: This study aimed to evaluate the long-term oncologic impact of anastomotic leakage, with particular focus on stage- and location-specific differences, in patients undergoing curative colorectal cancer surgery. The authors reported stage- and location-specific differences, with a potential adverse impact observed in patients with stage III rectal cancer.

However, several important potential confounding factors, such as nutritional status and comorbidities, were not assessed in the analysis. These variables may substantially influence both postoperative complications and long-term oncologic outcomes. Therefore, I would suggest reanalyzing the data after incorporating these important confounders to improve the validity and interpretability of the findings.

Reviewer #2: Thank you for the opportunity to review this retrospective single-centre study of 2,122 patients, assessing whether clinically significant anastomotic leakage (Grade B/C) affects long-term oncologic outcomes after curative colorectal cancer surgery. Overall, AL was not an independent predictor of OS or DFS on multivariable analysis. However, subgroup analyses suggested worse outcomes in stage III disease, particularly stage III rectal cancer, potentially related to delays in adjuvant chemotherapy. My comments are as follows:

1. The primary analysis was negative (AL not independently associated with OS or DFS). The positive findings are based on small subgroup analyses of only 63 leak patients and should be over-relied on. The manuscript would be strengthened by presenting these findings more cautiously and emphasising that the primary multivariable analysis was negative.

2. The manuscript's central conclusion is based on subgroup analyses stratified by stage and tumour location. However, formal interaction testing was not performed. Demonstration of statistical significance in one subgroup and non-significance in another does not establish a differential effect. The authors should perform interaction analyses (AL × stage and AL × tumour location) before concluding that the oncologic impact of AL differs according to disease stage or tumour location.

3. AL patients had significantly more rectal cancers. Residual confounding may remain despite adjustment, and methods such as propensity matching could strengthen the analysis. The authors should consider reporting separate leak rates for colon and rectal cancer.

4. The 2004–2018 timeframe spans major changes in surgery, perioperative care, neoadjuvant treatment and chemotherapy. Treatment era should be considered as a potential confounder.

5. The study evaluates clinically significant AL rather than all AL (grade A leaks excluded). This distinction should be stated consistently throughout the manuscript.

Reviewer #3: This is an interesting paper on the effects of anastomotic leak for colorectal cancer patients.

I have some points that I think should be addressed:

Introduction

Page 5 lines 6-8

Have there been any meta-analyses to support the points made here? If yes, they should be included.

Line 15

Please cite papers for the limited data on colon cancer

Methods

Page 7

A reference for the AJCC 7th edition should be included.

What was the definition of Stage 0 found in Table 4?

Results

Page 8

The number of colon and rectal patients should be stated, and the breakdown of how many colon or rectal patients had grade B or C AL

Why was 70 chosen as the dividing point for age? This should be explained

Why was 25 and over chosen for BMI? This should be explained, and whether the authors are using the WHO BMI guidelines for Asian patients

How were rectal cancer patients who had neoadjuvant chemoradiotherapy considered in the results?

Page 10

I think supplemental information and analyses for colon and rectal separately would be useful for readers.

Discussion/Conclusions

Given that patients with AL having issues after surgery for CRC is not new. I think a paragraph should be added explaining what this paper contributes to the literature and why it is important.

Limitations section

The low number of patients in some groups in the analyses is not included and should be.

Tables 2, 3, 4

What statistical analyses were done to create these p-values? This would be useful in the text and on the Table legends

Table 4

WD/MD and PD are not defined

General points

There are only 25 references in the paper, and none from 2025 or 2026. I recommend a search on PubMed for AL and CRC or AL and colon cancer, as I found numerous papers looking at AL and colon/CRC and postoperative mortality and morbidity.

I would be interested in the author's thoughts on whether this paper would be better as two separate papers on colon cancer and rectal cancer.

**********

what does this mean?). If published, this will include your full peer review and any attached files.

If you choose “no”, your identity will remain anonymous but your review may still be made public.

**Do you want your identity to be public for this peer review?**  For information about this choice, including consent withdrawal, please see our Privacy Policy

Reviewer #1: No

Reviewer #2: No

Reviewer #3: No

**********

[NOTE: If reviewer comments were submitted as an attachment file, they will be attached to this email and accessible via the submission site. Please log into your account, locate the manuscript record, and check for the action link "View Attachments". If this link does not appear, there are no attachment files.]

To ensure your figures meet our technical requirements, please review our figure guidelines: https://journals.plos.org/plosone/s/figures

You may also use PLOS’s free figure tool, NAAS, to help you prepare publication quality figures: https://journals.plos.org/plosone/s/figures#loc-tools-for-figure-preparation.

NAAS will assess whether your figures meet our technical requirements by comparing each figure against our figure specifications.

# Author comment: Author response to Decision Letter 1

**DOI:** 10.1371/journal.pone.0359416.r002

17 Aug 2026

● Reviewer #1:

This study aimed to evaluate the long-term oncologic impact of anastomotic leakage, with particular focus on stage- and location-specific differences, in patients undergoing curative colorectal cancer surgery. The authors reported stage- and location-specific differences, with a potential adverse impact observed in patients with stage III rectal cancer.

However, several important potential confounding factors, such as nutritional status and comorbidities, were not assessed in the analysis. These variables may substantially influence both postoperative complications and long-term oncologic outcomes. Therefore, I would suggest reanalyzing the data after incorporating these important confounders to improve the validity and interpretability of the findings.

Answer)

Thank you for this important comment. In the original analysis, ASA classification and BMI were included as general indicators of comorbidity and nutritional status. However, we agree that these measures alone may not adequately capture the potential confounding effects of underlying comorbidities and nutritional impairment. Therefore, we additionally evaluated cardiovascular disease, diabetes mellitus, chronic kidney disease, and preoperative hypoalbuminemia, defined as a serum albumin level of <3.5 g/dL within 30 days before surgery. These variables were assessed in the univariate survival analyses and were entered into the multivariable Cox regression models when the predefined criterion of p<0.20 was met. Clinically significant AL and treatment era were retained irrespective of their univariate significance.

After incorporating these additional variables and treatment era, clinically significant AL remained not independently associated with either OS (HR, 1.581; 95% CI, 0.923–2.707; p=0.095) or DFS (HR, 1.325; 95% CI, 0.812–2.161; p=0.260). The Methods, Results, Table 2, Table 5, and the location-specific supplementary analyses have been revised accordingly.

● Reviewer #2:

Thank you for the opportunity to review this retrospective single-centre study of 2,122 patients, assessing whether clinically significant anastomotic leakage (Grade B/C) affects long-term oncologic outcomes after curative colorectal cancer surgery. Overall, AL was not an independent predictor of OS or DFS on multivariable analysis. However, subgroup analyses suggested worse outcomes in stage III disease, particularly stage III rectal cancer, potentially related to delays in adjuvant chemotherapy. My comments are as follows:

1. The primary analysis was negative (AL not independently associated with OS or DFS). The positive findings are based on small subgroup analyses of only 63 leak patients and should be over-relied on. The manuscript would be strengthened by presenting these findings more cautiously and emphasising that the primary multivariable analysis was negative.

Answer)

We agree that the primary multivariable analyses did not demonstrate an independent association between clinically significant AL and either OS or DFS. In the revised analyses incorporating additional clinical variables and treatment era, AL remained not independently associated with OS (HR, 1.581; 95% CI, 0.923–2.707; p=0.095) or DFS (HR, 1.325; 95% CI, 0.812–2.161; p=0.260).

We therefore revised the Abstract, Results, Discussion, and Conclusions to emphasize the negative primary multivariable findings and to present the stage- and location-specific findings more cautiously. In addition, formal interaction analyses were performed, as suggested in Comment 2 below. Because these interaction tests did not demonstrate significant effect modification by either tumor stage or location, the subgroup findings are now described as exploratory observations rather than definitive evidence of differential oncologic effects. We have also emphasized the limited number of AL events and the resulting reduced statistical power of subgroup analyses in the Limitations section.

2. The manuscript's central conclusion is based on subgroup analyses stratified by stage and tumour location. However, formal interaction testing was not performed. Demonstration of statistical significance in one subgroup and non-significance in another does not establish a differential effect. The authors should perform interaction analyses (AL × stage and AL × tumour location) before concluding that the oncologic impact of AL differs according to disease stage or tumour location.

Answer)

Thank you for the important comment. We agree with the reviewer that statistical significance in one subgroup and non-significance in another does not, by itself, establish a differential effect. Accordingly, we performed formal interaction analyses using multivariable Cox proportional hazards models to evaluate whether the association between clinically significant AL and survival outcomes differed according to tumor stage or tumor location. Interaction terms between AL and tumor stage (AL × stage) and between AL and tumor location (AL × location) were evaluated separately for OS and DFS. Stage 0 was excluded from the stage interaction analysis because of its small sample size and clinical heterogeneity.

The interactions between AL and tumor stage were not statistically significant for either OS (p for interaction=0.132) or DFS (p for interaction=0.141). Likewise, there was no significant interaction between AL and tumor location for OS (p for interaction=0.775) or DFS (p for interaction=0.748). Although the stage III subgroup showed a significant adjusted association between AL and poorer OS (HR, 2.36; 95% CI, 1.27–4.36) and DFS (HR, 1.94; 95% CI, 1.10–3.41), the nonsignificant interaction tests indicate that these findings do not provide statistical evidence that the effect of AL differs according to tumor stage or location.

We have added the interaction analyses to the Methods and Results and present the findings in a new Table 6. The Abstract, Discussion, and Conclusions have also been revised so that the stage- and location-specific findings are interpreted as exploratory rather than as evidence of established effect modification.

3. AL patients had significantly more rectal cancers. Residual confounding may remain despite adjustment, and methods such as propensity matching could strengthen the analysis. The authors should consider reporting separate leak rates for colon and rectal cancer.

Answer)

We agree that the higher proportion of rectal cancer in the AL group could contribute to confounding. We have now explicitly reported the incidence of clinically significant AL according to tumor location: AL occurred in 23 of 1,351 patients with colon cancer (1.7%) and in 40 of 771 patients with rectal cancer (5.2%).

Tumor location was evaluated as a potential prognostic variable in the overall survival analyses and was entered into the multivariable model when it met the predefined univariate selection criterion. In addition, we performed separate multivariable Cox regression analyses for colon and rectal cancer and formal interaction analyses between AL and tumor location. The AL-by-location interaction was not significant for either OS (p for interaction=0.775) or DFS (p for interaction=0.748).

We also considered propensity score matching as suggested by the reviewer. However, because only 63 patients experienced clinically significant AL, propensity score matching would substantially reduce the effective sample size and statistical precision, particularly for the stage- and location-specific analyses. We therefore retained the full-cohort multivariable Cox regression as the primary approach and supplemented it with location-specific Cox regression and formal interaction analyses. The possibility of residual confounding from unmeasured factors has also been emphasized in the Limitations section.

4. The 2004–2018 timeframe spans major changes in surgery, perioperative care, neoadjuvant treatment and chemotherapy. Treatment era should be considered as a potential confounder.

Answer)

We agree that the long study period may have introduced temporal heterogeneity related to changes in surgical techniques, perioperative management, neoadjuvant treatment, and systemic chemotherapy. To address this potential confounding, we categorized the study period into three 5-year treatment eras (2004–2008, 2009–2013, and 2014–2018) according to the year of surgery and incorporated treatment era as an additional covariate in the revised multivariable Cox regression analyses.

After adjustment for treatment era, the association between anastomotic leakage and long-term oncologic outcomes remained essentially unchanged, and AL was not independently associated with either overall survival or disease-free survival. We have added the treatment-era variable to the Methods, Results, and relevant tables. We have also acknowledged in the Discussion that treatment practices evolved over the 15-year study period and that adjustment for calendar era may not fully reflect all changes in clinical practice.

5. The study evaluates clinically significant AL rather than all AL (grade A leaks excluded). This distinction should be stated consistently throughout the manuscript.

Answer)

We agree that our study specifically evaluated clinically significant AL rather than all grades of AL. In this study, AL was defined as grade B or C leakage according to the International Study Group of Rectal Cancer (ISREC) classification, whereas grade A leakage, which does not require active therapeutic intervention, was excluded.

Accordingly, we have revised the manuscript to consistently clarify that the AL group represents patients with ‘clinically significant anastomotic leakage (grade B or C)’. This terminology has been clarified in the overall manuscript and title.

● Reviewer #3:

This is an interesting paper on the effects of anastomotic leak for colorectal cancer patients.

I have some points that I think should be addressed:

Introduction

Page 5 lines 6-8

Have there been any meta-analyses to support the points made here? If yes, they should be included.

Answer)

We have added recent systematic reviews and meta-analyses to provide stronger evidence regarding the incidence of anastomotic leakage after colorectal cancer surgery. We have revised the Introduction accordingly and added the relevant references.

Line 15

Please cite papers for the limited data on colon cancer

Answer)

We agree that the statement regarding the relatively limited evidence for colon cancer should be supported by appropriate references. We have therefore added references to previous studies specifically evaluating the oncologic impact of anastomotic leakage after colon cancer surgery. We also revised the sentence to avoid overstating the paucity of available evidence, as follows:

“Previous studies on the long-term oncologic impact of AL have predominantly focused on rectal cancer, whereas relatively fewer studies have specifically evaluated patients with colon cancer.”

Methods

Page 7

A reference for the AJCC 7th edition should be included.

Answer)

We added a reference for the AJCC 7th edition.

What was the definition of Stage 0 found in Table 4?

Answer)

Thank you for this important comment. In the present study, Stage 0 comprised patients with pTisN0M0 disease after upfront surgery and those with ypT0N0M0 disease after NCRT. We have clarified this definition in the footnote of Table 4.

Furthermore, patients with stage 0 disease were included in the overall cohort because they met the study eligibility criteria. However, they were not included in the stage-specific survival analyses, which focused on stages I–III. This was because the number of patients with stage 0 disease was small (n=51, 2.4% of the entire cohort), with only two patients experiencing clinically significant anastomotic leakage, precluding a meaningful and statistically reliable stage-specific comparison. We have added this explanation to the methods section.

Results

Page 8

The number of colon and rectal patients should be stated, and the breakdown of how many colon or rectal patients had grade B or C AL

Answer)

We have revised the results section to provide the number of patients according to tumor location and the distribution of grade B and C AL according to tumor location. Among the 2,122 patients, 1,351 had colon cancer and 771 had rectal cancer. Of the 63 patients who developed clinically significant AL, 23 patients had colon cancer and 40 patients had rectal cancer. Among patients with colon cancer, 7 patients had grade B and 16 patients had grade C AL, whereas among those with rectal cancer, 11 patients had grade B and 29 patients had grade C AL. These data have been added to the results section.

Why was 70 chosen as the dividing point for age? This should be explained

Answer)

The cutoff of 70 years was selected primarily based on our institutional practice for preoperative risk assessment. At our institution, patients aged 70 years or older routinely undergo additional preoperative evaluation by both cardiology and pulmonology, regardless of the presence of underlying cardiopulmonary comorbidities. Therefore, age was categorized as <70 and ≥70 years to reflect this clinically relevant institutional criterion rather than on the basis of a data-driven cutoff. In addition, an age threshold of approximately 70 years has also been commonly used in previous studies and recommendations addressing older patients with colorectal cancer. We have clarified the rationale for this age cutoff in the methods section and added relevant reference.

Why was 25 and over chosen for BMI? This should be explained, and whether the authors are using the WHO BMI guidelines for Asian patients

Answer)

BMI was categorized as <25 and ≥25 kg/m², with BMI ≥25 kg/m² defined as obesity according to the Korean Society for the Study of Obesity guidelines. We have clarified the rationale for this BMI cutoff in the methods section and added relevant reference.

Additional revision:

While revising the manuscript, we also reviewed the cutoff used for operative time. Because the previous cutoff of 240 minutes lacked a clearly established clinical basis, operative time was reclassified using the cohort median of 150 minutes, and all relevant analyses were repeated. The Methods and relevant tables have been updated accordingly.

How were rectal cancer patients who had neoadjuvant chemoradiotherapy considered in the results?

Answer)

Thank you for this important comment. Rectal cancer patients who received neoadjuvant chemoradiotherapy (NCRT) were included in both the overall cohort and the rectal cancer subgroup analyses. For stage-specific analyses, postoperative pathologic stage was used; pTNM was applied to patients undergoing upfront surgery, whereas ypTNM was applied to patients receiving NCRT.

NCRT was also evaluated as a clinicopathologic variable in the rectal cancer-specific survival analyses. According to our predefined variable-selection criterion, NCRT was not entered into the multivariable OS model because its univariate p value was ≥0.20, whereas it was entered into the multivariable DFS model (univariate p=0.096) and was not independently associated with DFS after adjustment (HR, 1.182; 95% CI, 0.816–1.713; p=0.377).

We additionally reported the proportion of patients receiving NCRT in Table 2. Because NCRT was applicable only to patients with rectal cancer, the corresponding numbers and percentages were calculated among rectal cancer patients only. NCRT was not included as a covariate in the Cox regression analysis of the entire colorectal cancer cohort because its applicability was inherently dependent on tumor location.

Page 10

I think supplemental information and analyses for colon and rectal separately would be useful for readers.

Answer)

Separate subgroup analyses for colon and rectal cancer are presented in Supplementary Tables 1 and 2 (newly S1 Table, and S2 Table), respectively. These analyses include univariate log-rank and multivariable Cox regression analyses for OS and DFS. In the rectal cancer subgroup, rectal cancer-specific variables, including NCRT, diverting ileostomy, and anastomotic level from the anal verge (<5 cm vs. ≥5 cm), were additionally evaluated.

In the revised analyses, clinically significant AL was not independently associated with either OS or DFS in the colon cancer subgroup (OS: HR, 1.301; 95% CI, 0.532–3.182; p=0.565; DFS: HR, 1.141; 95% CI, 0.468–2.782; p=0.773) or in the rectal cancer subgroup (OS: HR, 1.340; 95% CI, 0.661–2.718; p=0.416; DFS: HR, 1.362; 95% CI, 0.741–2.502; p=0.320). The Results section has been expanded to summarize these location-specific findings and the significant independent prognostic factors identified in each subgroup.

Discussion/Conclusions

Given that patients with AL having issues after surgery for CRC is not new. I think a paragraph should be added explaining what this paper contributes to the literature and why it is important.

Answer)

We agree that the adverse short-term consequences of AL after colorectal cancer surgery are already well established. The contribution of the present study is therefore not simply to demonstrate that AL is harmful, but to provide a comprehensive evaluation of its association with long-term oncologic outcomes within a single colorectal cancer cohort, incorporating overall multivariable analyses, stage- and location-stratified analyses, and formal interaction testing.

Our revised analyses showed that clinically significant AL was not independently associated with OS or DFS in the overall cohort. Although poorer survival was observed among patients with stage III disease, particularly in the rectal cancer subgroup, formal interaction analyses did not demonstrate significant effect modification by either tumor stage or location. Thus, our findings provide a more cautious interpretation of the apparent subgroup patterns and demonstrate that statistical differences observed within individual subgroups should not be considered evidence of a differential AL effect without formal interaction testing.

In addition, the study evaluates both colon and rectal cancer within a consistent analytic framework and examines clinically relevant factors that may contribute to poorer outcomes after AL, including delays in the initiation of adjuvant chemotherapy among patients with stage III disease. We have revised the Discussion and Conclusions to clarify these contributions while explicitly acknowledging the exploratory nature of the subgroup findings.

Limitations section

The low number of patients in some groups in the analyses is not included and should be.

Answer)

We have expanded the Limitations section to address this issue. Although the overall study population was large, only 63 patients experienced clinically significant AL, and the number of AL events became considerably smaller after stratification by tumor stage and location. This may have reduced statistical power, widened the confidence intervals of subgroup estimates, and increased the risk of type II error, including limited power to detect interaction effects. Therefore, the stage- and location-specific subgroup findings are now explicitly described as exploratory and requiring validation in larger multicenter cohorts.

Tables 2, 3, 4

What statistical analyses were done to create these p-values? This would be useful in the text and on the Table legends

Answer)

We have clarified the statistical methods used for comparisons in Tables 2–4. Categorical variables were compared using Pearson’s chi-square test or Fisher’s exact test, as appropriate according to the expected cell counts. This information has been added to the statistical analysis section of the methods and to the legends of Tables 2–4.

Table 4

WD/MD and PD are not defined

Answer)

We have clarified the definitions of WD/MD and PD directly in Table 4. WD/MD denotes well/moderately differentiated tumors, and PD denotes poorly differentiated tumors. The table and its footnote have been revised accordingly.

General points

There are only 25 references in the paper, and none from 2025 or 2026. I recommend a search on PubMed for AL and CRC or AL and colon cancer, as I found numerous papers looking at AL and colon/CRC and postoperative mortality and morbidity.

Answer)

We performed an updated PubMed search using terms including “anastomotic leakage,” “colorectal cancer,” “colon cancer,” “mortality,” and “morbidity,” with particular attention to studies published in 2025 and 2026. Based on this updated search, we added recent publications relevant to the definition, reporting, and clinical consequences of AL, including the 2025 CoReAL reporting framework by Heuvelings et al., the 2025 study by Gosavi et al. evaluating morbidity and non-oncologic mortality after AL following colon cancer resection, and the 2026 study by Müller et al. evaluating long-term survival after AL in colon cancer. The Introduction and Discussion have been updated accordingly.

I would be interested in the author's thoughts on whether this paper would be better as two separate papers on colon cancer and rectal cancer.

Answer)

Thank you for this thoughtful comment. We agree that colon and rectal cancers differ in several important clinical and treatment-related aspects, and we carefully considered whether they should be evaluated separately.

However, we believe that retaining both tumor locations within a single study is particularly appropriate for the present research question because it enables direct comparison of the association between AL and outcomes according to tumor location within the same cohort and analytic framework. Importantly, the revised study now includes a formal AL-by-tumor-location interaction analysis, which directly evaluates whether the association of AL with survival differs between colon and rectal cancer. Such a comparison would not be possible in the same manner if the cohort were divided into two independent studies.

In addition, the number of patients with clinically significant AL was relatively small, particularly after stratification (23 patients with colon cancer and 40 with rectal cancer). Dividing the cohort into two independent studies would further reduce the statistical power of each analysis, particularly for stage-specific comparisons.

For these reasons, we believe that presenting colon and rectal cancer together, while providing detailed location-specific analyses, allows a more informative assessment of the association between AL and oncologic outcomes than separating the data into two individual papers.

Thank you again for your insightful comments and suggestions. We sincerely appreciate the time and effort you devoted to reviewing our manuscript and providing constructive comments.

Sincerely,

Jeong-Heum Baek, M.D., Ph.D.

(On behalf of all co-authors)

Attachment Submitted filename: PLOSONE_AL_Response to Reviewers.docx

# Peer review: Decision Letter 1

**DOI:** 10.1371/journal.pone.0359416.r003

**Authors:** Chong-Chi Chiu

15 Sep 2026

Long-term oncologic outcomes associated with clinically significant anastomotic leakage after curative colorectal cancer surgery: stage- and location-stratified analyses

PONE-D-26-21823R1

Dear Dr. Baek,

We’re pleased to inform you that your manuscript has been judged scientifically suitable for publication and will be formally accepted for publication once it meets all outstanding technical requirements.

Within one week, you’ll receive an e-mail detailing the required amendments. When these have been addressed, you’ll receive a formal acceptance letter and your manuscript will be scheduled for publication.

An invoice will be generated when your article is formally accepted. Please note, if your institution has a publishing partnership with PLOS and your article meets the relevant criteria, all or part of your publication costs will be covered. Please make sure your user information is up-to-date by logging into Editorial Manager at Editorial Manager® and clicking the ‘Update My Information' link at the top of the page. For questions related to billing, please contact billing support.

If your institution or institutions have a press office, please notify them about your upcoming paper to help maximize its impact. If they’ll be preparing press materials, please inform our press team as soon as possible -- no later than 48 hours after receiving the formal acceptance. Your manuscript will remain under strict press embargo until 2 pm Eastern Time on the date of publication. For more information, please contact onepress@plos.org.

Kind regards,

Chong-Chi Chiu

Academic Editor

PLOS One

Reviewers' comments:

Reviewer's Responses to Questions

**Comments to the Author**

Reviewer #1: All comments have been addressed

Reviewer #3: All comments have been addressed

**********

2. Is the manuscript technically sound, and do the data support the conclusions?

Reviewer #1: Yes

Reviewer #3: Yes

**********

3. Has the statistical analysis been performed appropriately and rigorously?

Reviewer #1: Yes

Reviewer #3: Yes

**********

4. Have the authors made all data underlying the findings in their manuscript fully available?

The PLOS Data policy

Reviewer #1: Yes

Reviewer #3: Yes

**********

5. Is the manuscript presented in an intelligible fashion and written in standard English?

Reviewer #1: Yes

Reviewer #3: Yes

**********

Reviewer #1: Overall, the revised manuscript is substantially improved, and the major concerns raised previously have been satisfactorily addressed. I have no further major comments or suggestions regarding the scientific content or presentation of the manuscript.

Reviewer #3: Thank you for all your responses to my comments and the time taken to address everything. I think the paper is much improved.

**********

what does this mean?). If published, this will include your full peer review and any attached files.

If you choose “no”, your identity will remain anonymous but your review may still be made public.

**Do you want your identity to be public for this peer review?**  For information about this choice, including consent withdrawal, please see our Privacy Policy

Reviewer #1: No

Reviewer #3: No

**********

# Editor report: Acceptance letter

**DOI:** 10.1371/journal.pone.0359416.r004

**Authors:** Chong-Chi Chiu

PONE-D-26-21823R1

PLOS One

Dear Dr. Baek,

I'm pleased to inform you that your manuscript has been deemed suitable for publication in PLOS One. Congratulations! Your manuscript is now being handed over to our production team.

At this stage, our production department will prepare your paper for publication. This includes ensuring the following:

* All references, tables, and figures are properly cited

* All relevant supporting information is included in the manuscript submission,

* There are no issues that prevent the paper from being properly typeset

You will receive further instructions from the production team, including instructions on how to review your proof when it is ready. Please keep in mind that we are working through a large volume of accepted articles, so please give us a few days to review your paper and let you know the next and final steps.

Lastly, if your institution or institutions have a press office, please let them know about your upcoming paper now to help maximize its impact. If they'll be preparing press materials, please inform our press team within the next 48 hours. Your manuscript will remain under strict press embargo until 2 pm Eastern Time on the date of publication. For more information, please contact onepress@plos.org.

You will receive an invoice from PLOS for your publication fee after your manuscript has reached the completed accept phase. If you receive an email requesting payment before acceptance or for any other service, this may be a phishing scheme. Learn how to identify phishing emails and protect your accounts at https://explore.plos.org/phishing.

If we can help with anything else, please email us at customercare@plos.org.

Thank you for submitting your work to PLOS One and supporting open access.

Kind regards,

PLOS One Editorial Office Staff

on behalf of

Professor Chong-Chi Chiu

Academic Editor

PLOS One
